# EPCR-PAR1 biased signaling regulates perfusion recovery and neovascularization in peripheral ischemia

**DOI:** 10.1172/jci.insight.157701

**Published:** 2022-07-22

**Authors:** Magdalena L. Bochenek, Rajinikanth Gogiraju, Stefanie Großmann, Janina Krug, Jennifer Orth, Sabine Reyda, George S. Georgiadis, Henri M. Spronk, Stavros Konstantinides, Thomas Münzel, John H. Griffin, Philipp Wild, Christine Espinola-Klein, Wolfram Ruf, Katrin Schäfer

**Affiliations:** 1 Center for Thrombosis and Hemostasis and; 2 Department of Cardiology, Cardiology I, University Medical Center Mainz, Mainz, Germany.; 3 Department of Vascular Surgery, University Hospital Alexandroupolis, Alexandroupolis, Greece.; 4 CARIM School for Cardiovascular Disease, Maastricht University, Maastricht, The Netherlands.; 5 Department of Cardiology, Democritus University of Thrace, Alexandroupolis, Greece.; 6 German Center for Cardiovascular Research, Rhine-Main Site.; 7 Department of Molecular Medicine, The Scripps Research Institute, La Jolla, California, USA.; 8 Department of Cardiology, Cardiology III, University Medical Center Mainz, Mainz, Germany.; 9 Immunology and Microbiology, The Scripps Research Institute, La Jolla, California, USA.

**Keywords:** Angiogenesis, Vascular Biology, Cardiovascular disease, Coagulation, Proteases

## Abstract

Blood clot formation initiates ischemic events, but coagulation roles during postischemic tissue repair are poorly understood. The endothelial protein C receptor (EPCR) regulates coagulation, as well as immune and vascular signaling, by protease activated receptors (PARs). Here, we show that endothelial EPCR-PAR1 signaling supports reperfusion and neovascularization in hindlimb ischemia in mice. Whereas deletion of PAR2 or PAR4 did not impair angiogenesis, EPCR and PAR1 deficiency or PAR1 resistance to cleavage by activated protein C caused markedly reduced postischemic reperfusion in vivo and angiogenesis in vitro. These findings were corroborated by biased PAR1 agonism in isolated primary endothelial cells. Loss of EPCR-PAR1 signaling upregulated hemoglobin expression and reduced endothelial nitric oxide (NO) bioavailability. Defective angiogenic sprouting was rescued by the NO donor DETA-NO, whereas NO scavenging increased hemoglobin and mesenchymal marker expression in human and mouse endothelial cells. Vascular specimens from patients with ischemic peripheral artery disease exhibited increased hemoglobin expression, and soluble EPCR and NO levels were reduced in plasma. Our data implicate endothelial EPCR-PAR1 signaling in the hypoxic response of endothelial cells and identify suppression of hemoglobin expression as an unexpected link between coagulation signaling, preservation of endothelial cell NO bioavailability, support of neovascularization, and prevention of fibrosis.

## Graphical abstract

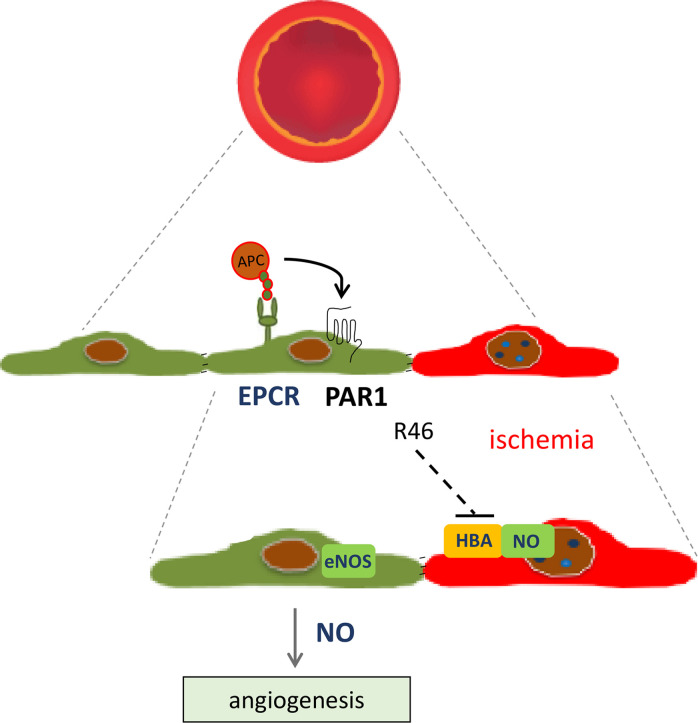

## Introduction

Acute or chronic obstruction of arterial blood flow causes ischemia as an important and often life-threatening consequence of atherosclerosis and its thrombotic complications. Early reperfusion is the preferred therapeutic strategy to prevent ischemic tissue loss, fibrotic scar formation, and mortality. Platelet aggregation inhibitors and anticoagulants, as well as interventional approaches, are effective (1) and recommended (2) for the restoration of blood flow in acute and subacute ischemia. In chronic ischemia, neovascularization processes involving angiogenesis and arteriogenesis are induced by the presence of hypoxia and changes in blood flow, but endogenous mechanisms of blood flow restoration are often impaired in patients with peripheral artery disease (PAD).

Endothelial cells play an important role in the prevention of intravascular thrombus formation, and endothelial dysfunction is critically involved in defective thrombus resolution and the development of fibrosis (3). The anticoagulant properties of the endothelium are mediated primarily by thrombomodulin (TM), a multidomain type-1 transmembrane glycoprotein constitutively expressed on the luminal surface of endothelial cells (4). TM binds the serine protease thrombin and thereby alters thrombin substrate specificity from fibrinogen cleavage to greatly enhance the activation of protein C, a serine protease with potent anticoagulant activity via proteolytic inactivation of coagulation factor Va (FVa) and FVIIIa (5). Activation of protein C on the surface of endothelial cells is further enhanced by its binding to endothelial protein C receptor (EPCR) (6), a type 1 transmembrane protein expressed on endothelial cells of large blood vessels and also on microvascular endothelial cells (MECs).

In addition to its anticoagulant activity, activated protein C (aPC) mediates cytoprotective signaling through activation of protease-activated receptors (PARs), a family of G-protein–coupled receptors expressed on endothelial cells, platelets, immune cells, and other cell types (7). aPC induced PAR1 signaling mediates antiapoptotic and antiinflammatory activities (8) and prevents thrombin-induced leukocyte or endothelial cell activation (9). One determinant of the cytoprotective effects of aPC is the noncanonical proteolytic cleavage of PAR1 at Arg46 creating an alternative tethered ligand with biased signaling specificity (10). In contrast, the canonical cleavage site of thrombin resulting in proinflammatory PAR1 signaling occurs at Arg41 (11). In addition, PAR1 cleavage by platelet-derived calpains can amplify endothelial dysfunction by proteolytic shedding of EPCR, as shown in patients with risk factors for cardiovascular disease (12).

Here, we employed a series of genetic mouse models and primary endothelial cells, as well as vascular specimens of patients with ischemic PAD to delineate the roles of EPCR and signaling via PARs in vascular regeneration and restoration of tissue perfusion following ischemic injury. We identified a crucial role for EPCR-PAR1 biased signaling in regulating endothelial nitric oxide (NO) homeostasis and the restoration of blood flow in peripheral ischemia with potential implications for improvements in therapeutic neovascularization strategies in patients with PAD.

## Results

### EPCR is upregulated on endothelial cells during postischemic neovascularization.

In C57BL/6N WT mice subjected to unilateral hindlimb ischemia, we observed high levels of EPCR expression on CD31^+^ endothelial cells in ischemic hindlimbs at days 7 and 14 (Supplemental Figure 1, A and B; supplemental material available online with this article; https://doi.org/10.1172/jci.insight.157701DS1). In contrast, the number of CD31^+^ cells staining for thrombin (Supplemental Figure 1, C and D) or the number of lectin^+^ cells staining for the platelet marker CD41 (Supplemental Figure 1, E and F) was significantly increased only at later time points — i.e., at days 21 and 28. Fibrinogen^+^ endothelial cells were also significantly increased at day 28 (Supplemental Figure 1, G and H), indicating progressive coagulation activation during ischemic revascularization and repair. The observed temporarily separated and early increase of EPCR expression on endothelial cells raised the question whether the aPC pathway might serve functions beyond the control of blood coagulation during ischemic vessel regeneration.

### EPCR deletion with a Tie2.Cre driver results in impaired blood flow recovery and hindlimb neovascularization following ischemia.

**Figure 1:**
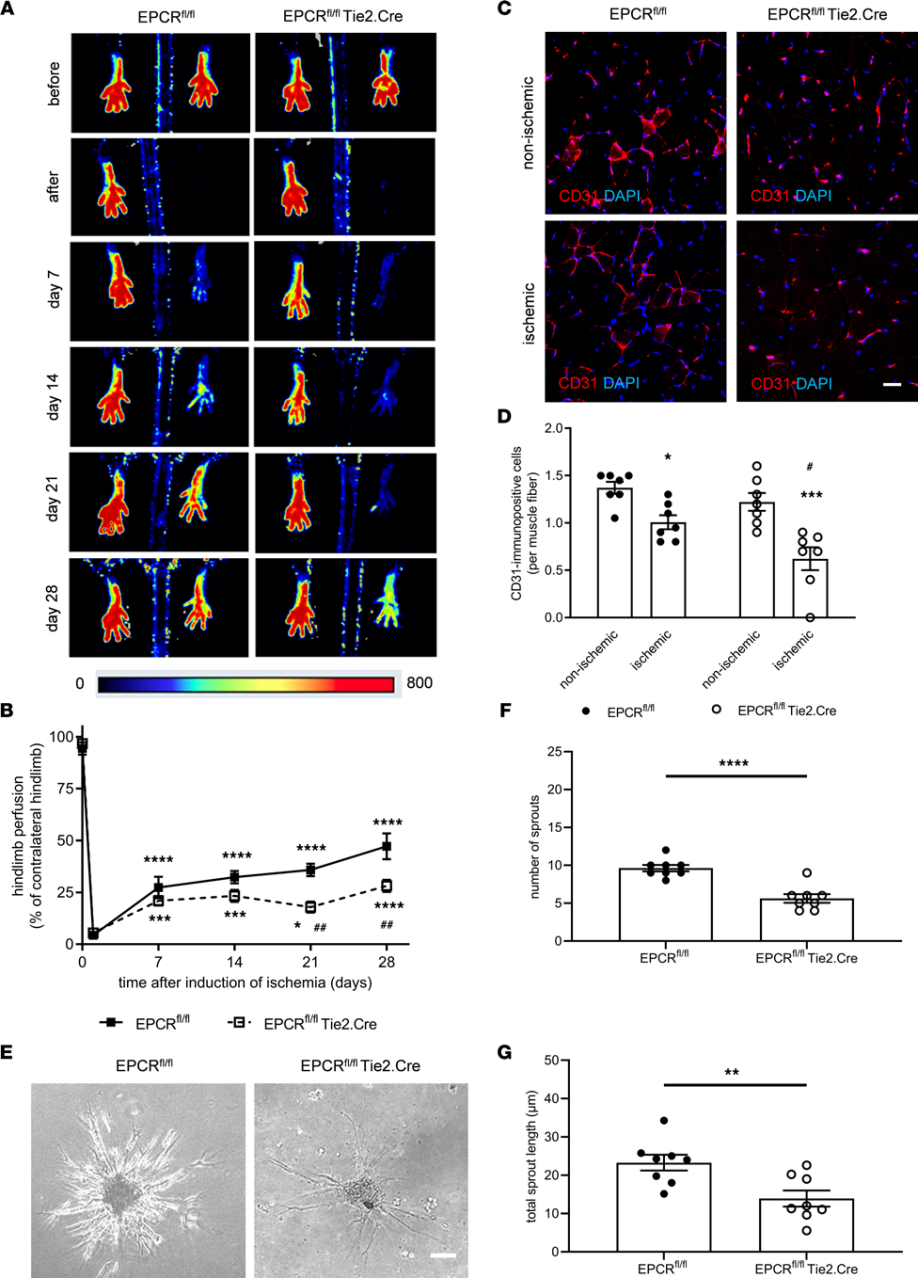
Reperfusion and new vessel formation following ischemia in mice lacking EPCR in Tie2-expressing cells. (**A**) Representative laser Doppler perfusion images in EPCR^fl/fl^ Tie2.Cre mice (*n* = 11) and EPCR^fl/fl^ littermate controls (*n* = 10) immediately before and after, as well as on, day 7, day 14, day 21, and day 28 after induction of hindlimb ischemia. For technical reasons, 1 animal in each group could not be imaged at day 21. (**B**) Quantitative analysis of the laser signal (expressed as % of the contralateral, nonischemic site). *P < 0.05, ***P < 0.001, and ****P < 0.0001 versus values immediately after surgery; ^##^*P* < 0.01 versus EPCR^fl/fl^ controls at the same time point; 2-way ANOVA, Sidak’s multiple-comparison test. (**C**) Representative immunofluorescence microscopy images after visualization of CD31 expression (red) in nonischemic and ischemic hindlimbs of EPCR^fl/fl^ Tie2.Cre mice and control littermates. DAPI^+^ cell nuclei appear blue. Scale bar: 10 μm. (**D**) Quantitative analysis of CD31-immunopositive cells per muscle fiber; *n* = 7 biological replicates. *P < 0.05 and ***P < 0.001 versus nonischemic control muscle; ^#^*P* < 0.05 versus EPCR^fl/fl^ littermates; 2-way ANOVA, Sidak’s multiple-comparison test. (**E**) Representative bright-field images of CD31^+^ endothelial cells isolated from EPCR^fl/fl^ and EPCR^fl/fl^ Tie2.Cre littermate mice and subjected to the spheroid assay; *n* = 4 biological replicates per 2 experimental repeats. Scale bar: 10 μm. (**F** and **G**) Quantitative analysis of the number of sprouts (**F**) and total length of the sprouts (**G**) migrated out of the spheroids. **P < 0.01 and ****P < 0.0001 versus EPCR^fl/fl^ littermate control mice. Data were analyzed using Student’s *t* test.

To directly test the role of EPCR in endothelial cells, we generated EPCR^fl/fl^ mice and deleted EPCR using the constitutive pan–endothelial cell Tie2.Cre driver line targeting all vascular endothelial cells throughout embryogenesis and adulthood (13). Immunofluorescence confocal microscopy of lower hindlimb muscles and Western blot analysis of isolated cells confirmed the absence of EPCR in endothelial cells from EPCR^fl/fl^ Tie2.Cre mice. The majority of EPCR immunosignal colocalized with CD31 — and not with F4/80 in EPCR^fl/fl^ littermate controls (Supplemental Figure 2, A–D). Reperfusion was significantly reduced in EPCR^fl/fl^ Tie2.Cre mice compared with littermate EPCR^fl/fl^ controls at days 21 and 28 after ischemia induction (Figure 1, A and B). These findings were confirmed by quantification of CD31-immunopositive endothelial cells using confocal microscopy (Figure 1, C and D) or flow cytometry of hindlimb muscle cell suspensions (Supplemental Figure 3A). Deletion of EPCR also reduced sprouting angiogenesis of isolated endothelial cells in the spheroid angiogenesis assays (Figure 1, E–G), as well as in the aortic ring assay (Supplemental Figure 3, B–D).

Although TM is important for regulating thrombin activity intravascularly, TM mutant mice (TM^ProPro^) with decreased thrombin binding (14) showed unaltered hindlimb reperfusion when analyzed on the same genetic background as EPCR^fl/fl^ Tie2.Cre mice (Supplemental Figure 4, A and B). The genes for TM and EPCR are located on the same chromosome in close proximity, and TM^ProPro^ mice were originally generated by targeting in 129/SvPAS embryonic stem cells. On a mixed genetic background, the polymorphic EPCR allele of the targeted cell segregates with the TM^ProPro^ allele, resulting in markedly higher EPCR expression in mouse lung homogenates typical for 129/SvPAS mice (Supplemental Figure 4C) and in primary endothelial cells when TM^ProPro^ mice were compared with WT littermates on a B6-129/SvPAS mixed background (Supplemental Figure 4, D and E). Note that the TM^ProPro^ allele on a pure C57BL/6 background did not affect EPCR expression. Interestingly, the overexpression of EPCR led to a significantly increased reperfusion of the hindlimb (Supplemental Figure 4F). Moreover, a cross of TM^ProPro^ mice on a C57BL/6N background with 129/SvPas mice resulted in markedly enhanced reperfusion, and these F1 mice were indistinguishable from the mixed B6-129/SvPAS background TM^ProPro^ mice from day 14 onward (Supplemental Figure 4G). Thus, genetic variability in endothelial cell EPCR expression was associated with improved reperfusion after hindlimb ischemia.

EPCR also plays an important role in myeloid cell coagulation signaling (15), and constitutive Tie2.Cre during embryogenesis deletes genes in hematopoietic cells (16). However, deletion of EPCR in EPCR^fl/fl^ LysM.Cre mice did not impair reperfusion relative to littermate controls (Supplemental Figure 5, A and B), indicating that the phenotype of EPCR^fl/fl^ Tie2.Cre mice was primarily caused by EPCR deletion in endothelial and not myeloid cells. EPCR supports FXa-dependent signaling through PAR2 (17), but neither PAR2^–/–^ nor PAR4^–/–^ mice showed a significant reduction in reperfusion compared with C57BL/6N control mice examined in parallel (Supplemental Figure 5, C and D). We therefore focused our following analyses on the role of PAR1 signaling in new vessel formation.

### PAR1 deficiency and PAR1 resistance to aPC cleavage impair hindlimb reperfusion and angiogenic sprouting.

**Figure 2:**
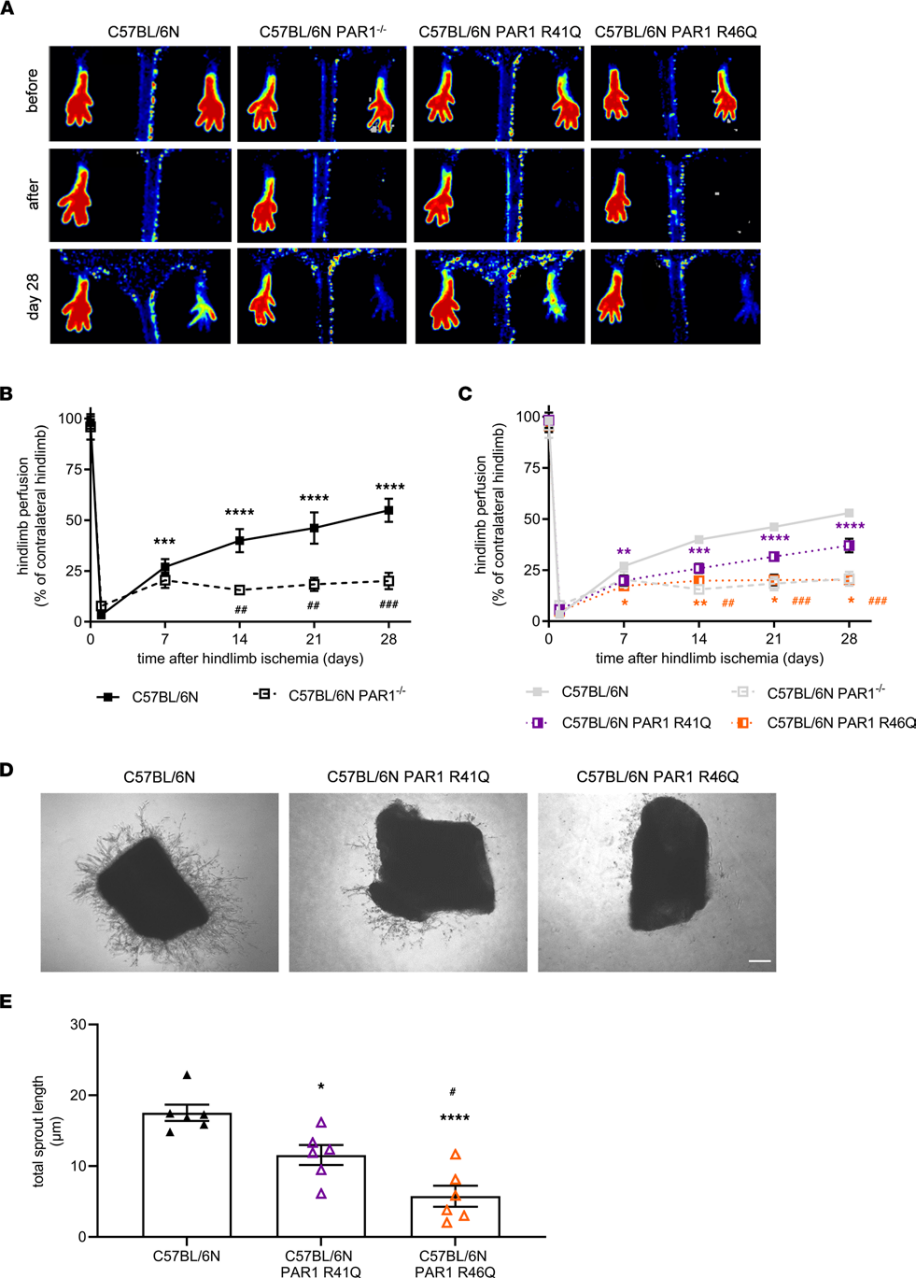
Reperfusion and angiogenic sprout formation in mice with global PAR1 deficiency or with R41Q or R46Q point mutations in the PAR1 gene. (**A**) Representative laser Doppler perfusion images in C57BL/6N control mice (first column), C57BL/6N PAR1^–/–^ (second column), C57BL/6N PAR1 R41Q (third column), and C57BL/6N PAR1 R46Q mice (fourth column) immediately before and after, as well as on, day 28 after induction of hindlimb ischemia are shown. (**B** and **C**) Summary of the quantitative analysis of the laser signal (expressed as % of the contralateral, nonischemic site) in C57BL/6N control mice (*n* = 12) and C57BL/6N PAR1^–/–^ (*n* = 9, *n* = 6 after day 14) (**B**) or in C57BL/6N PAR1 R41Q (*n* = 13, *n* = 9 after day 14; purple) and C57BL/6N PAR1 R46Q (*n* = 13, *n* = 9 after day 14; orange (**C**). Data from **B** are shown in gray for comparison as well. *P < 0.05, **P < 0.01, ***P < 0.001 and ****P < 0.0001 versus values immediately after surgery; ^##^*P* < 0.01 and ^###^*P* < 0.001 versus control mice at the same time point; 2-way ANOVA, Sidak’s multiple-comparison test. (**D**) Representative bright-field images of aortic rings isolated from C57BL/6N control mice and from C57BL/6N PAR1 R41Q and C57BL/6N PAR1 R46Q. Scale bar: 10 μm. (**E**) Quantitative analysis of total length of sprouts migrated from aortic rings of C57BL/6N control mice, C57BL/6N PAR1 R41Q (purple), and C57BL/6N PAR1 R46Q (orange). *P < 0.05 and ****P < 0.0001 versus C57BL/6N; ^#^*P* < 0.05 versus C57BL/6N PAR1 R41Q; *n* = 3 biological replicates per 2 experimental repeats. One-way ANOVA followed by Sidak’s multiple comparisons.

In contrast to PAR2- and PAR4-deficient mice, serial laser Doppler perfusion imaging (LDPI) measurements revealed significantly reduced reperfusion after induction of hindlimb ischemia in mice with global PAR1 deficiency (PAR1^–/–^) compared with C57BL/6N control mice examined in parallel, beginning at day 14 and continuing until day 28 (Figure 2, A and B). Of note, genetic PAR1 deletion (Supplemental Figure 6, A and B) was not associated with mRNA expression changes of other PARs in endothelial cells isolated from lungs (versus C57BL/6N controls *P* = 0.1464 for PAR2 and *P* = 0.3424 for PAR3). Interestingly, the expression of EPCR was significantly downregulated upon PAR1 deletion (Supplemental Figure 6, C and D), whereas EPCR deletion did not affect the expression of PAR1 (Supplemental Figure 6, E and F).

Thrombin as well as EPCR-dependent FXa and FVIIa signaling are mediated by cleavage at PAR1 R41 (18), whereas cytoprotective signaling by aPC is mediated by cleavage at R46, which generates a tethered ligand with biased PAR1 agonistic activity (10). Of note, PAR1 mRNA expression levels were not altered in cleavage-insensitive PAR1 R41Q or PAR1 R46Q mutant mice (Supplemental Figure 6, B, G, and H). Induction of hindlimb ischemia in mice genetically modified to express the aPC-resistant PAR1 R46Q mutant (19) resulted in significantly lower reperfusion compared with mice carrying a mutation in the canonical PAR1 cleavage site — i.e. PAR1 R41Q mutant, which did not significantly differ from C57BL/6N controls (Figure 2, A and C). Endothelial cell density was not different in nonischemic muscle in these mutant mice relative to WT, but the reduction of endothelial cells in ischemic hindlimbs was much more pronounced in PAR1^–/–^, PAR1 R41Q, and PAR1 R46Q mice when compared with control C57BL/6N mice (Supplemental Figure 7, A and B).

Angiogenic sprout formation ex vivo was also impaired in aortic rings isolated from PAR1 R46Q mutant mice, whereas it was reduced to a significantly lesser extent in aortic rings from PAR1 R41Q mutant mice (Figure 2, D and E). Supporting a role for endothelial biased PAR1 signaling during angiogenesis, the formation of angiogenic sprouts from aortic rings (Supplemental Figure 8, A–C) or by primary endothelial cell spheroids (Supplemental Figure 8, D–F) from C57BL/6N mice was enhanced in response to agonist peptides corresponding to the N-terminus of PAR1 exposed by aPC cleavage (TR47) — but not by scrambled control peptides or peptides mimicking PAR1 cleaved by thrombin (TR41). Thus, biased agonist PAR1 signaling is the major driver for postischemic neovascularization.

### Inhibition of coagulation activation by tissue factor FVIIa impairs endothelial sprouting and neovascularization.

**Figure 3:**
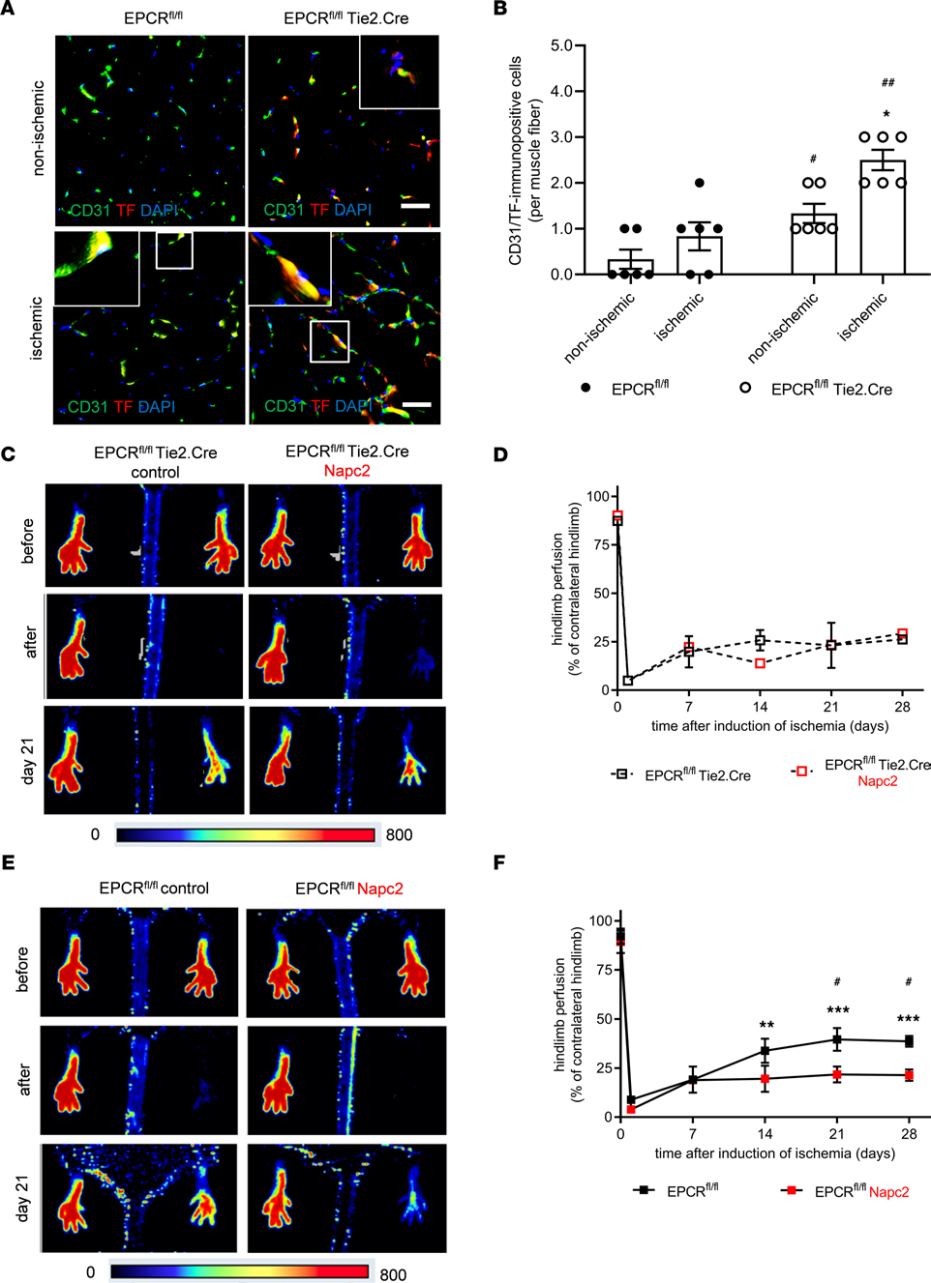
Role of tissue factor in EPCR proangiogenic signaling. (**A** and **B**) Representative high resolution immunofluorescence images (**A**) and quantitative analysis (**B**) after staining of ischemic and nonischemic hindlimb muscles of EPCR^fl/fl^ Tie2.Cre mice and EPCR^fl/fl^ littermate controls with antibodies against TF (red) and CD31 (green). Two-way ANOVA, Sidak’s multiple-comparison test. *P < 0.05 versus the nonischemic, contralateral hindlimb; ^#^*P* < 0.05 and ^##^*P* < 0.01 versus EPCR^fl/fl^ control littermate mice; *n* = 6 biological replicates. (**C** and **E**) Representative laser Doppler perfusion images in EPCR^fl/fl^ Tie2.Cre (*n* = 3 control, *n* = 2 NAPc2 treatment) (**C**) and littermate EPCR^fl/fl^ control mice (*n* = 3) (**E**) immediately before and after, as well as on, day 21 after induction of hindlimb ischemia, treated with PBS as a control or NAPc2 (0.5 mg/kg body weight; directly after surgery and afterward every 2 days). (**D** and **F**) Quantitative analysis of the laser signal (expressed as % of the contralateral, nonischemic site) for EPCR^fl/fl^ Tie2.Cre (**D**) and EPCR^fl/fl^ littermate controls (**F**). **P < 0.01 and ***P < 0.001 versus values immediately after surgery; ^#^*P* < 0.05 versus untreated controls at the same time point; 2-way ANOVA, Sidak’s multiple-comparison test.

Our data indicate that coagulation activation occurred during neovascularization (Supplemental Figure 1), and the coagulation initiator tissue factor (TF) is known to regulate angiogenic processes (20). Immunofluorescence microscopy demonstrated increased numbers of TF-immunopositive cells in hindlimb muscles following induction of ischemia, and the increased expression was particularly striking in EPCR^fl/fl^ Tie2.Cre mice (Figure 3, A and B). High-resolution fluorescence microscopy localized TF immunosignals primarily to CD31^+^ endothelial cells in ischemic muscles, whereas within ischemic hindlimbs, only a few SMA^+^ myofibroblast-like cells (Supplemental Figure 9, A and B) or F4/80^+^ macrophages (Supplemental Figure 9, C and D) expressed TF.

We, therefore, asked whether TF played a role in locally activating the coagulation cascade to generate thrombin, which in turn resulted in EPCR-aPC-PAR1 signaling. We used the nematode-derived TF inhibitor nematode anticoagulant peptide c2 (NAPc2), which inhibits TF-FVIIa in a FX/Xa dependent manner (21) and thereby is a potent inhibitor of TF-dependent thrombin generation. NAPc2 did not influence the already defective blood flow recovery observed in EPCR^fl/fl^ Tie2.Cre mice (Figure 3, C and D), indicating that increased thrombin generation downstream of the elevated TF levels was not contributing to the impaired postischemic angiogenesis observed in this mouse line. Rather, NAPc2 phenocopied the effects of EPCR deletion in Tie2.Cre-expressing cells on the neovascularization response to ischemia in vivo (Figure 3, E and F). NAPc2 also reduced angiogenic sprout formation ex vivo (Supplemental Figure 10, A and B) and in vitro (Supplemental Figure 10, C and D). These findings are in line with prior data demonstrating suppression of TF-dependent angiogenesis (22) and suggest that TF-initiated thrombin (23) and downstream aPC generation are crucial for mediating postischemic neovascularization dependent on EPCR-PAR1 signaling in endothelial cells.

### EPCR-PAR1 signaling controls endothelial NO bioavailability through regulation of hemoglobin.

To better understand the mechanisms of defective angiogenesis in EPCR-PAR1 signaling–deficient mice, we performed explorative mRNA transcript profiling of primary endothelial cells isolated from EPCR^fl/fl^ Tie2.Cre and from PAR1 mutant mice. These data suggest that hemoglobin, but not myoglobin mRNA transcript levels, was increased more than 5-fold in endothelial cells lacking EPCR or expressing the aPC cleavage–resistant PAR1 R46Q (not shown). Previous studies have shown hemoglobin expression at the myoendothelial junction of endothelial cells in resistance arteries and implicated it in the regulation of endothelial NO bioavailability and vascular tone (24, 25), suggesting hemoglobin upregulation as a possible mechanism underlying the impaired angiogenesis in these mutant mice.

**Figure 4:**
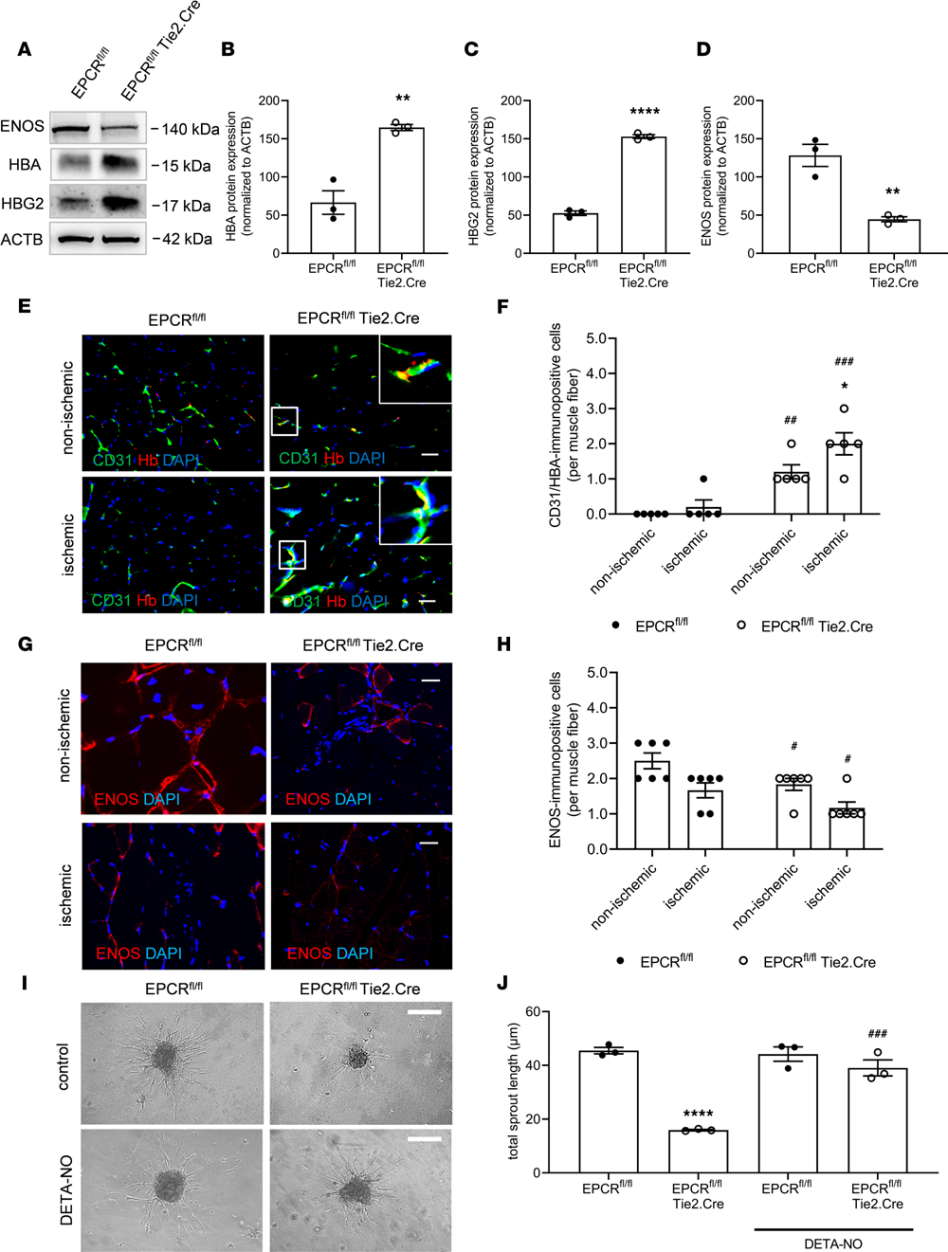
EPCR-dependent hemoglobin expression and its effects on nitric oxide bioavailability and function. (**A**–**D**) Representative western blot membranes and their quantification showing HBA, HBG2 (methemoglobin), and eNOS protein levels in murine primary endothelial cells isolated from lungs of EPCR^fl/fl^ Tie2.Cre mice and EPCR^fl/fl^ littermate controls; *n* = 3 biological replicates. **P < 0.01 and ****P < 0.0001 versus EPCR^fl/fl^ mice; unpaired Student’s *t* test. (**E** and **F**) Representative confocal images and the results of the quantitative analysis after visualization of HBA (red) expression in CD31^+^ endothelial cells (green) on cryopreserved cross sections isolated at day 28 after ischemia; *n* = 5 biological replicates. DAPI^+^ cell nuclei appear blue. Scale bars: 10 μm. Note that myoglobin expression was not detected in endothelial cells in EPCR^fl/fl^ Tie2.Cre mice and their littermate controls. (**G** and **H**) Representative confocal microscopy images and quantification of cryopreserved cross sections of nonischemic and ischemic hindlimbs of EPCR^fl/fl^ Tie2.Cre mice and EPCR^fl/fl^ littermate controls stained using antibodies against eNOS (red). DAPI^+^ cell nuclei appear blue. Scale bars: 10 μm; *n* = 3 biological replicates per 2 experimental repeats; ^#^*P* < 0.05 versus EPCR^fl/fl^ control littermate mice using 2-way ANOVA, Sidak’s multiple-comparison test. (**I** and **J**) Representative microscopy brightfield images and quantification of total sprout length in control and DETA-NO–treated primary endothelial cell spheroids from EPCR^fl/fl^ Tie2.Cre mice and EPCR^fl/fl^ littermate controls; *n* = 3 biological replicates. ***P < 0.001 versus EPCR^fl/fl^ littermate control mice; ^###^*P* < 0.001 versus untreated cells belonging to the same group; 1-way ANOVA, Sidak’s multiple-comparison test.

Although RBC parameters were unchanged in EPCR- and PAR1-deficient mice (Supplemental Table 1), Western blotting confirmed a significant increase in endothelial hemoglobin subunit α (HBA) expression in primary endothelial cells isolated from EPCR^fl/fl^ Tie2.Cre relative to EPCR^fl/fl^ littermate control mice (Figure 4, A and B). Moreover, increased expression of the reduced form of hemoglobin, methemoglobin (HBG2), was observed in cells isolated from EPCR^fl/fl^ Tie2.Cre mice (Figure 4, A and C), and conversely, protein levels of endothelial NO synthase (eNOS) were reduced (Figure 4, A and D). Immunofluorescence confocal microscopy confirmed strong endothelial hemoglobin expression in EPCR^fl/fl^ Tie2.Cre mice, both at baseline and even more pronounced following ischemia (Figure 4, E and F). In addition, the number of cells expressing eNOS was significantly reduced (Figure 4, G and H). Accordingly, the number of endothelial cells reacting with the fluorescent NO indicator DAF-2 DA was significantly reduced in ischemic and nonischemic hindlimbs of mice lacking EPCR (Supplemental Figure 11A). Of note, at this late time point after ischemia (day 28), Western blot analysis did not reveal any differences in eNOS phosphorylation (at Ser^1177^; not shown). These data are in line with increased hemoglobin-mediated NO scavenging in endothelial cells lacking EPCR.

We, therefore, analyzed the functional significance of reduced NO bioavailability in EPCR^fl/fl^ Tie2.Cre mice. Addition of the NO donor diethylenetriamine/NO (DETA-NO) to isolated primary EPCR^fl/fl^ Tie2.Cre endothelial cells completely rescued the defective sprouting in the spheroid assay (Figure 4, I and J) and also restored the impaired sprouting capacity of aortic rings from EPCR^fl/fl^ Tie2.Cre mice (Supplemental Figure 11, B and C). In line with a functional importance of EPCR expression levels, 129Sv/Pas C57BL/6 TM^Pro/Pro^ mice with increased expression of EPCR (Supplemental Figure 12, A and B) showed higher levels of eNOS (Supplemental Figure 12, A and C) and significantly lower levels of methemoglobin (Supplemental Figure 12, A and D). Levels of HBA were not significantly changed (Supplemental Figure 12E).

**Figure 5:**
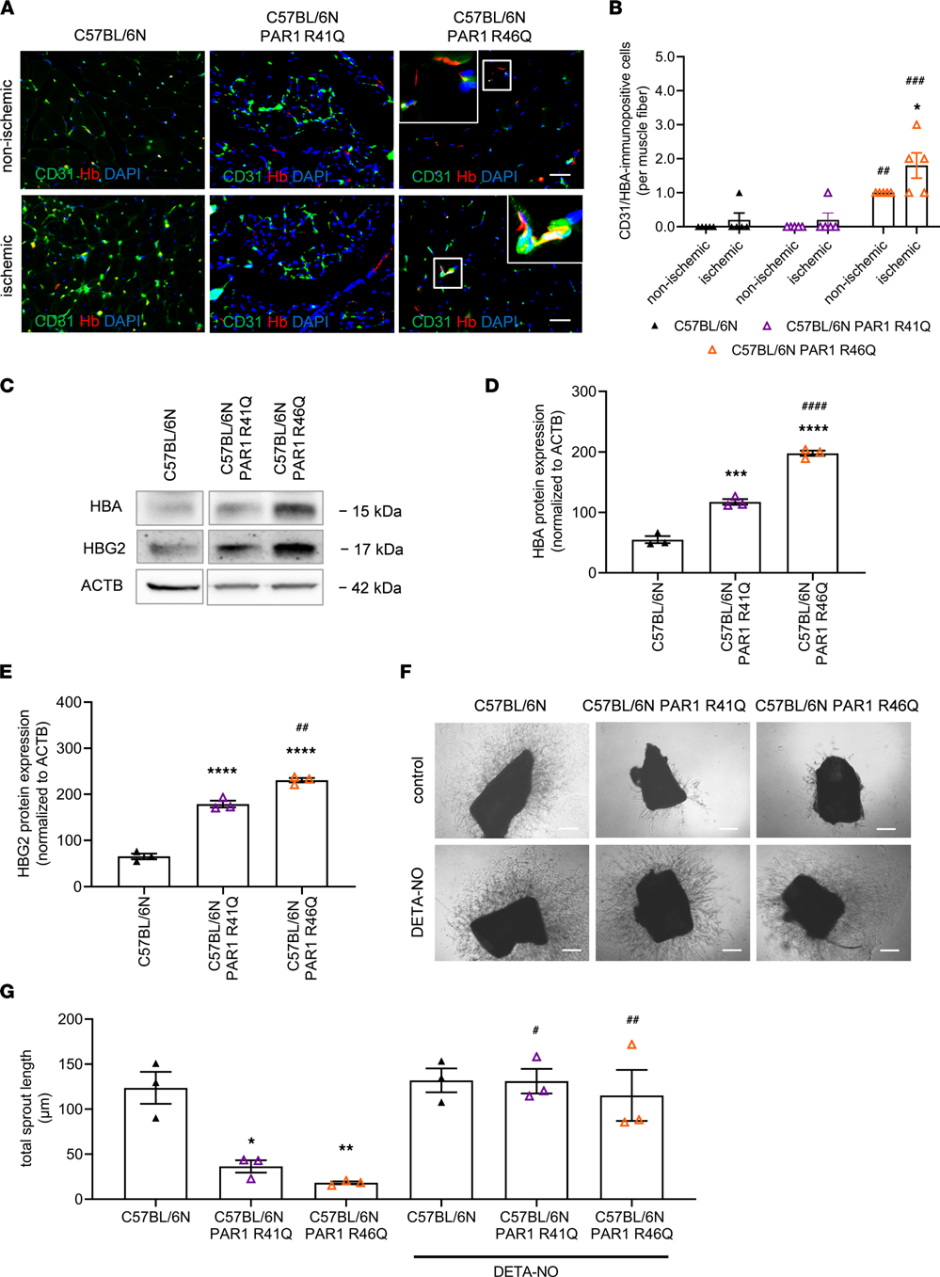
Endothelial hemoglobin expression and NO-dependent angiogenesis in PAR1 mutant mice. (**A** and **B**) Representative confocal immunofluorescence microscopy images and quantitative analysis after visualization of HBA (red) expression in CD31^+^ endothelial cells (green) on cryopreserved cross sections isolated from mice at day 28 after ischemia. DAPI^+^ cell nuclei appear blue. Scale bars: 10 μm. Two-way ANOVA, Sidak’s multiple comparison; *n* = 5 biological replicates. *P < 0.05 versus nonischemic hindlimb; ^##^*P* < 0.01 and ^###^*P* < 0.001 versus C57BL/6N control mice. (**C**–**E**) Representative Western blots and quantification of HBA and HBG2 (methemoglobin) protein levels in primary lung endothelial cells isolated from C57BL/6N, C57BL/6N PAR1 R41Q, and C57BL/6N PAR1 R46Q mutant mice; *n* = 3 biological replicates. Samples were run on the same blot. Nonadjacent lanes are indicated by an empty space between them. One-way ANOVA, Sidak’s multiple comparison. ***P < 0.001 and ****P < 0.0001 versus C57BL/6N; ^##^*P* < 0.01 and ^####^*P* < 0.0001 versus C57BL/6N PAR1 R41Q. Angiogenic sprout formation from aortic rings of C57BL/6N, C57BL/6N PAR1 R41Q, and C57BL/6N PAR1 R46Q mutant mice with and without DETA-NO (100 μM). (**F** and **G**) Representative findings and quantitative analysis are shown; *n* = 3 biological replicates. Scale bars: 100 μm. *P < 0.05 and **P < 0.01 versus C57BL/6N; ^#^*P* < 0.05 and ^##^*P* < 0.01 for DETA-NO versus untreated aortic rings of the same group. One-way ANOVA, Sidak’s multiple-comparison test.

Immunofluorescence confocal microscopy demonstrated increased hemoglobin expression also in PAR1 R46Q mutant mice, predominantly in ischemic and, to a lesser extent, in contralateral nonischemic hindlimbs (Figure 5, A and B). Significantly increased endothelial HBA and HBG2 protein levels were confirmed in PAR1 R46Q mutant mice compared with C57BL/6N control mice and to PAR1 R41Q mutants, as shown by Western blot analysis (Figure 5, C–E). Importantly, exogenous NO supplementation significantly enhanced endothelial angiogenic sprout formation from aortic rings (Figure 5, F and G) and primary endothelial cells (Supplemental Figure 13, A and B) isolated from PAR1 mutant mice. Thus, maintaining NO bioavailability during angiogenic processes is a primary function of endothelial EPCR-PAR1 signaling. Total erythrocyte number, hematocrit, and hemoglobin levels were unchanged in EPCR- and PAR1-deficient mice (Supplemental Table 1).

### Reduced endothelial EPCR levels and NO bioavailability in patients with PAD.

**Figure 6:**
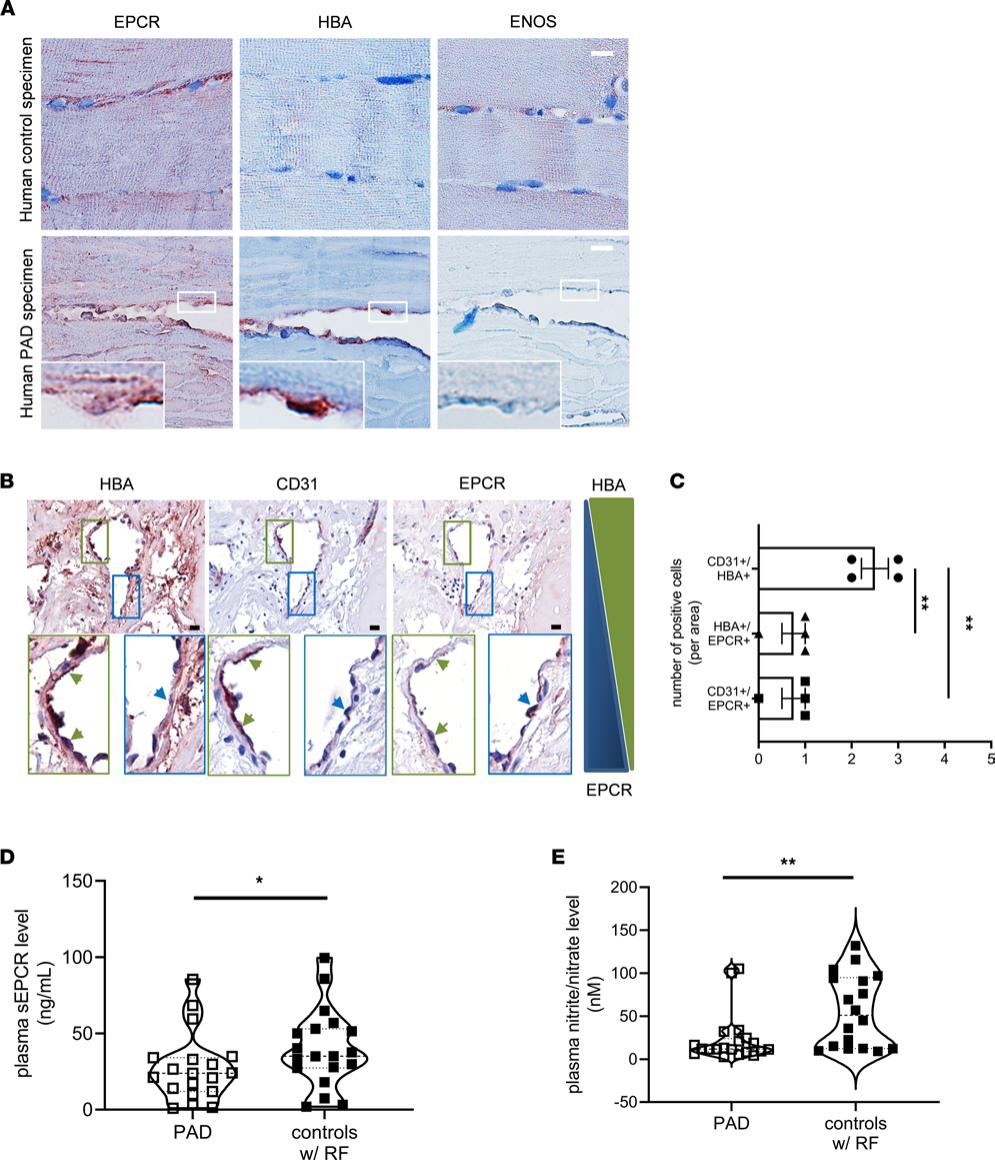
Endothelial EPCR and hemoglobin expression and soluble EPCR plasma levels in patients with chronic peripheral artery ischemia. (**A**) Representative IHC images of human skeletal muscle specimens from nonischemic controls (top panel) and patients with peripheral artery disease (PAD) showing expression of EPCR, HBA, and eNOS^+^ endothelial cells; *n* = 4 biological replicates. Scale bars: 10 μm. (**B**) Representative IHC images of human PAD specimens showing expression of HBA, CD31, and EPCR on serial cross sections. Scale bars: 10 μm (upper row). Lower row shows enlarged areas of interest. Images are taken with 200× (upper row) and 400× (lower row) magnification. (**C**) Results of quantitative analysis of the number of positive cells residing in vascular specimens isolated from patients with PAD; *n* = 4 biological replicates. **P < 0.01; 1-way ANOVA, Bonferroni’s multiple-comparison test. (**D**) Plasma levels of soluble EPCR (sEPCR) in 19 patients with PAD and 19 age- and sex-matched individuals with similar risk factors (RF) and no PAD diagnosis (controls with RF). (**E**) Plasma levels of nitrite/nitrate in patients with PAD and age-and sex-matched controls. *P < 0.05 and **P < 0.01. Student’s *t* test.

We used biobanked material from patients with ischemic PAD to evaluate the clinical relevance of our findings. Histological analysis of vascular specimens from patients with PAD showed elevated endothelial hemoglobin and reduced EPCR expression in CD31^+^ endothelial cells compared with skeletal muscle biopsies from nonischemic control patients (Figure 6, A–C). Moreover, plasma levels of soluble EPCR (Figure 6D) and nitrite/nitrate (Figure 6E) were significantly reduced in a cohort of patients with PAD (*n* = 19) compared with age- and sex-matched individuals (*n* = 19) with similar cardiovascular risk factors and no diagnosis of PAD. The clinical and functional characteristics of the study population for these latter analyses are shown in Supplemental Tables 2 and 3.

### Hypoxia is associated with EPCR internalization and upregulation of NRF2.

**Figure 7:**
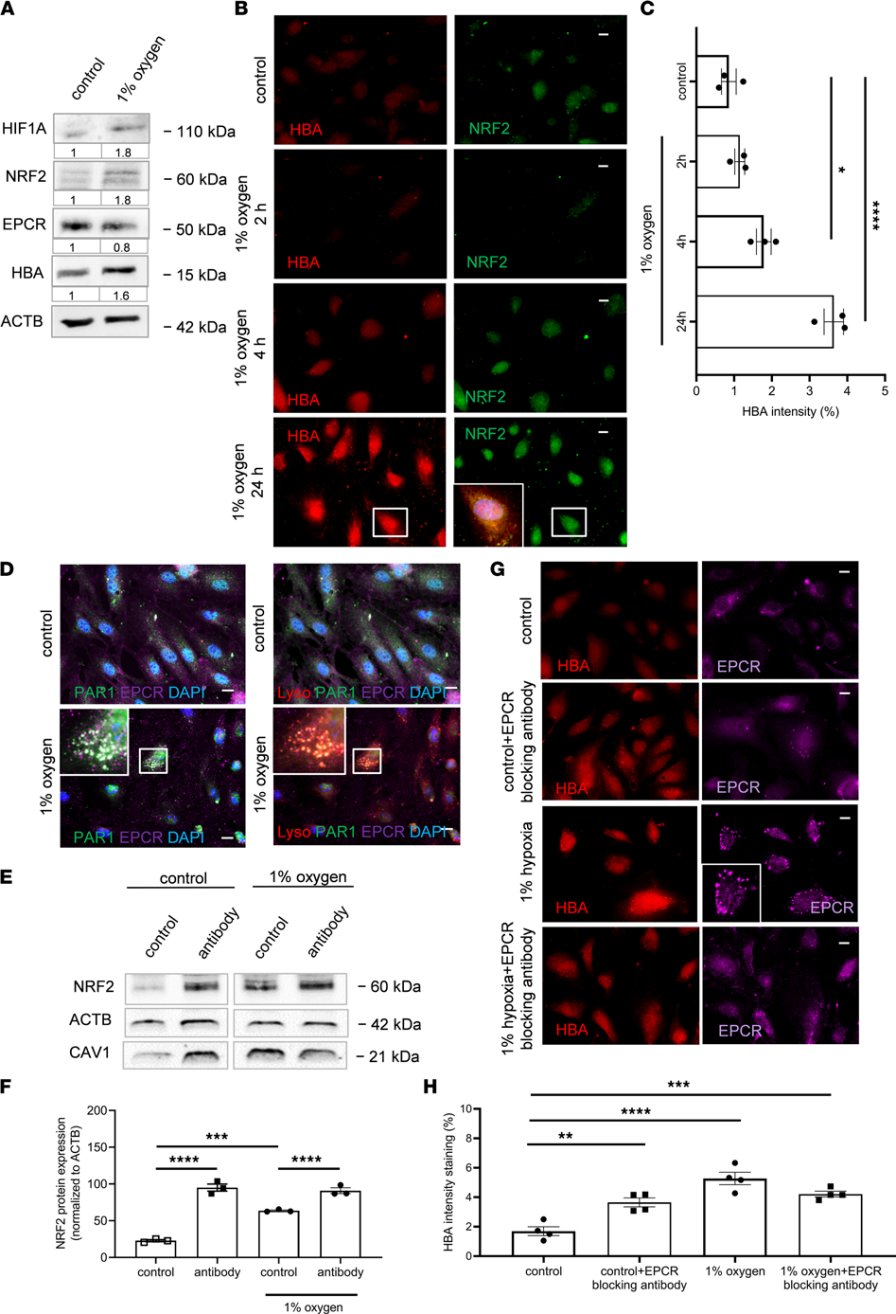
Effect of hypoxia on endothelial hemoglobin and EPCR expression. (**A**) Representative Western blot of human cardiac microvascular endothelial cells cultured under hypoxia (1% oxygen) showing HIF1A, NRF2, EPCR, HBA, and ACTB as a protein loading control; *n* = 3 biological replicates. Values under each blot image indicate fold changes versus results in control cells (as determined by normalization to the loading control run on the same blot). (**B** and **C**) Representative immunofluorescent images and quantification of human microvascular endothelial cells cultured under hypoxia at different time points, as indicated, and stained for HBA (red) and NRF2 (green). Small image in 24-hour NRF2 panel shows overlay image of the cell. *P < 0.05 and ****P < 0.0001 versus control calculated using 1-way ANOVA, Bonferroni’s multiple-comparison test. (**D**) Representative immunofluorescent high-resolution images showing human microvascular endothelial cells cultured under hypoxia (1% oxygen) and stained for PAR1 (green) and EPCR (purple) at the left panel or for PAR1 (green), EPCR (purple), and lysosomal tracker (Lyso; red) at the right panel; *n* = 3 biological replicates. Scale bars: 10 μm. (**E** and **F**) Western blot and quantification analysis of human microvascular endothelial cells cultured under hypoxia (1% oxygen) showing NRF2, caveolin-1, and β-actin (ACTB) as a loading control; *n* = 3 biological replicates. ***P < 0.001 and ****P < 0.0001; 1-way ANOVA, Bonferroni’s multiple-comparison test. (**G** and **H**) Representative immunofluorescent images and quantification analysis showing human microvascular endothelial cells cultured under hypoxia (1% oxygen), treated with EPCR-blocking antibody (20 μg/mL), as indicated, and stained for HBA (red) and EPCR (purple). Scale bars: 10 μm. **P < 0.01, ***P < 0.001, and ****P < 0.0001 versus control cells; 1-way ANOVA, Bonferroni’s multiple-comparison test.

We next asked whether the chronic hypoxia in clinical PAD might contribute to deregulation of EPCR signaling in endothelial cells. We cultured HCMECs under conditions of 1% oxygen and demonstrated not only increased protein levels of hypoxia-induced factor-α (HIF1A), but also of HBA and of nuclear factor erythroid 2–related factor 2 (NRF2), a transcription factor known to activate hemoglobin gene expression (26–28). Remarkably, protein levels of EPCR were reduced (Figure 7A). Immunofluorescence microscopy analysis at different time points revealed strong cellular HBA immunosignals, together with increased NRF2 expression after 24 hours of incubation (Figure 7, B and C). At this time point, high-resolution imaging revealed an increased appearance of EPCR and PAR1 immunostaining in the perinuclear region marked with lysosomal tracker of endothelial cells exposed to hypoxia (Figure 7D), indicating hypoxia-induced internalization of EPCR and PAR1.

We next tested the function of cell-surface EPCR by blocking ligand binding with specific antibodies. EPCR-blocking antibodies induced the upregulation of NRF2 already under normoxic conditions but had no additive effect on NRF2 levels in cells exposed to hypoxia (Figure 7, E and F). A similar pattern was observed for protein levels of caveolin-1, an eNOS antagonist coexpressed with EPCR in lipid rafts (29, 30) (Figure 7E). Immunofluorescence signals of HBA also increased in HCMECs treated with EPCR blocking antibodies under normoxic conditions, and anti-EPCR had no additional effect in cells cultured in 1% oxygen (Figure 7, G and H). These data support a model in which chronic hypoxia induces a functional inactivation of cell-surface EPCR activity by stimulating EPCR and PAR1 internalization, resulting in increased NO scavenging in the context of hemoglobin upregulation.

In HCMECs, pharmacological NO scavenging using PTIO or inhibition of intracellular NO signaling using ODQ, a competitive and heme-site specific antagonist of the intracellular NO receptor soluble guanylyl cyclase (sGC), significantly increased *HBA* and hemoglobin subunit β (*HBB*) mRNA levels, whereas gene expression decreased in response to the NO donor DETA-NO (Supplemental Figure 14). These data support the relevance of a negative reciprocal interaction between intracellular NO and hemoglobin levels in human endothelial cells.

### Impaired NO signaling increases the expression of mesenchymal markers in endothelial cells and promotes myofibroblast accumulation and fibrosis.

**Figure 8:**
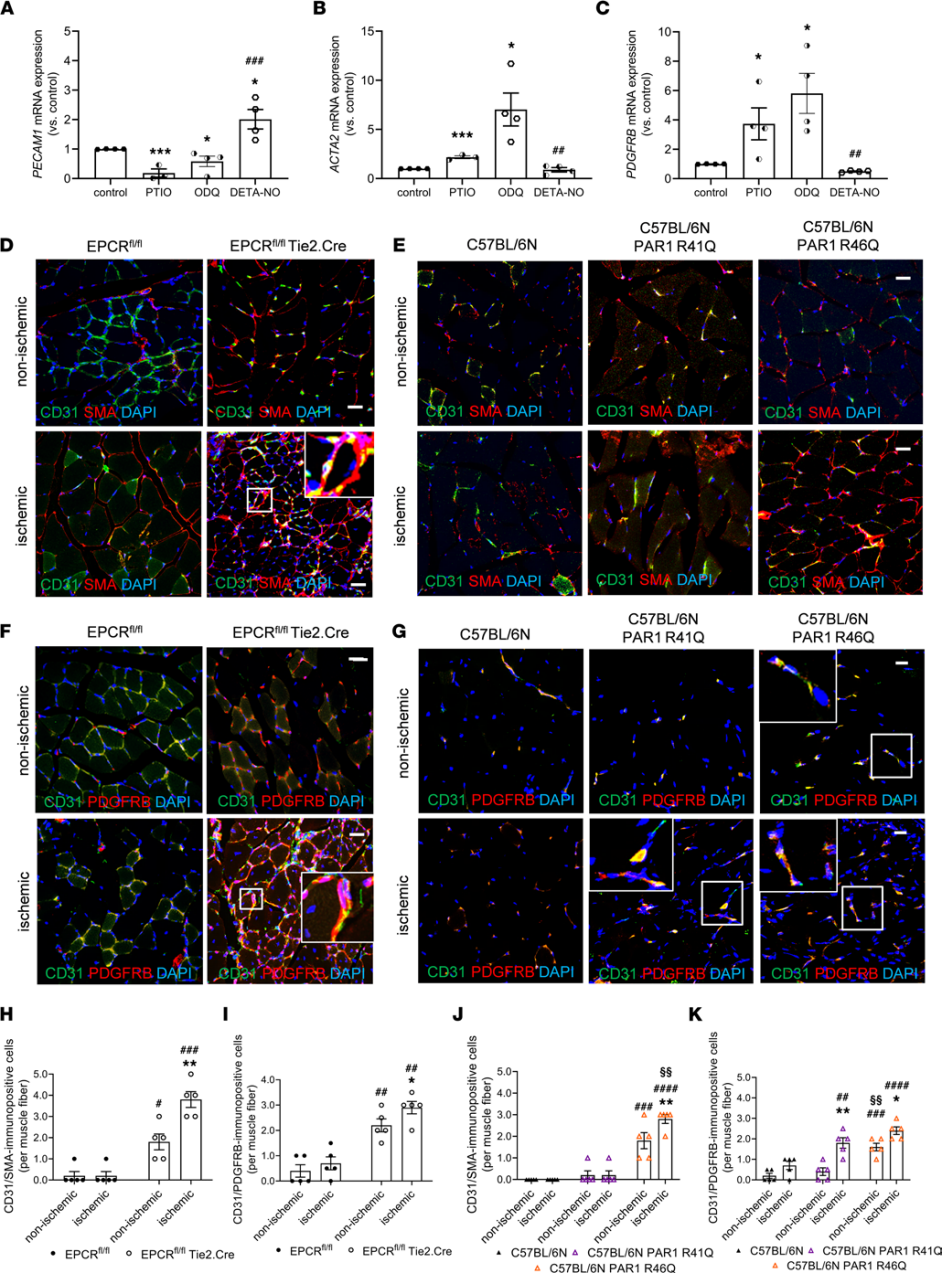
Mesenchymal marker expression in endothelial cells and hindlimbs in the presence of altered NO signaling. (**A**–**C**) Human microvascular endothelial cells were treated with PTIO (100 μM), ODQ (50 μM), or the NO donor DETA-NO (100 μM), and changes in the mRNA transcript levels of platelet-endothelial cell adhesion molecule (*PECAM1*) (**A**), smooth muscle α-actin (*ACTA2*) (**B**), and platelet-derived growth factor receptor-β (*PDGFRB*) (**C**) were examined 4 days later using qPCR; *n* = 3-4 biological replicates. One-way ANOVA, Sidak’s multiple-comparison test. *P < 0.05 and ***P < 0.001 versus control; ^##^*P* < 0.01 and ^###^*P* < 0.001 versus ODQ-treated cells. (**D**, **E**, **H**, and **J**) Representative confocal images and quantitative analysis after immunostaining of SMA (red) and CD31 expression (green) in ischemic (**D** and **H**) and nonischemic hindlimb muscles of EPCR^fl/fl^ Tie2.Cre mice and EPCR^fl/fl^ littermate control mice or C57BL/6N, C57BL/6N PAR1 R41Q, and C57BL/6N PAR1 R46Q mutant mice (**E** and **J**); *n* = 5 biological replicates. DAPI^+^ cell nuclei appear blue. Scale bars: 10 μm. (**F**, **G**, **I**, and **K**) Representative confocal images and (**I**) quantitative analysis after immunostaining of PDGFRB (red) and CD31 expression (green) in ischemic and nonischemic hindlimb muscles of EPCR^fl/fl^ Tie2.Cre and EPCR^fl/fl^ control littermate mice (**F** and **I**) or C57BL/6N, C57BL/6N PAR1 R41Q, and C57BL/6N PAR1 R46Q mutant mice at day 28 after ischemia (**G** and **K**); *n* = 5 biological replicates. DAPI^+^ cell nuclei appear blue. Scale bars: 10 μm. *P < 0.05 and **P < 0.01 versus nonischemic, contralateral hindlimb; ^##^*P* < 0.01, ^###^*P* < 0.001, and ^####^*P* < 0.0001 versus C57BL/6N control mice; ^§§^*P* < 0.01 versus C57BL/6N PAR1 R41Q mice. Two-way ANOVA, Sidak’s multiple-comparison test.

We hypothesized that impaired EPCR-PAR1 signaling leading to increased hemoglobin-mediated NO scavenging may affect angiogenesis by altering endothelial cell phenotypes. Quantitative PCR (qPCR) analysis of HCMECs showed that mRNA transcript levels of the pan–endothelial marker CD31 (*PECAM1*) increased in response to the NO donor DETA-NO and decreased in the presence of the NO scavenger PTIO or the NO signaling inhibitor ODQ (Figure 8A). Treatment of HCMECs with PTIO and ODQ also resulted in increased mRNA expression of the mesenchymal markers SMA (*ACTA2*; Figure 8B) and PDGFR-β (*PDGFRB*; Figure 8C), whereas mRNA levels of both markers significantly decreased in response to DETA-NO.

In line with the increased expression of hemoglobin in endothelial cells of EPCR^fl/fl^ Tie2.Cre and PAR1 R46Q mice, both strains showed increased numbers of CD31^+^ cells that were also immunopositive for SMA (Figure 8, D and E) or PDGFRB (Figure 8, F and G). Coexpression of SMA and PDGFRB in CD31^+^ cells further increased in ischemic hindlimb muscles 28 days after femoral artery ligation in both mutant strains (Figure 8, H–K). Moreover, EPCR^fl/fl^ Tie2.Cre displayed increased amounts of interstitial collagen in ischemic and in contralateral hindlimbs (Supplemental Figure 15, A and B), and fibrosis was also increased in ischemic muscles of PAR1 R46Q mice (Supplemental Figure 15, C and D). In contrast, and in line with the expression analysis of isolated endothelial cells, PAR1 R41Q mice showed no increased abundance of SMA and PDGFRB expression in CD31^+^ cells in the nonischemic muscle, but PDGFRB expression (Figure 8K) and fibrosis (Supplemental Figure 15, C and D) were increased in the ischemic tissue. Because thrombin was detectable particularly at later stages of revascularization, these data indicate that canonical cleavage of PAR1 also contributed to the hypoxic response in peripheral ischemia, in line with the observed slight reduction of neovascularization in PAR1 R41Q mice (Figure 2C).

## Discussion

In this study, we identify a critical role for EPCR-dependent biased PAR1 signaling in regenerative angiogenesis following arterial ischemia in mice and implicate EPCR-PAR1 signaling–mediated regulation of NO bioavailability and scavenging by hemoglobin downstream of EPCR-PAR1 signaling as a crucial mechanism preventing impaired postischemic tissue remodeling. We provide evidence that blood flow recovery and neovascularization in the unilateral hindlimb ischemia model depend on the presence of PAR1, but not on PAR2 or PAR4. Analysis of mice with targeted point mutations of the PAR1 Arg^46^ aPC cleavage site or stimulation with biased PAR1 agonist peptides indicate that angiogenesis is primarily driven by aPC-mediated PAR1 activation. Mechanistically, we demonstrate that genetic deletion of EPCR or defective PAR1 signaling are associated with increased expression of hemoglobin and its reduced form, methemoglobin, in endothelial cells, resulting in decreased NO bioavailability. Increased endothelial hemoglobin expression is also found in vascular specimens of patients with PAD with concomitant evidence for decreased soluble EPCR levels and NO bioavailability in plasma samples. These clinical data corroborate the preclinically delineated role of EPCR-PAR1 biased signaling during regeneration following peripheral ischemia.

Thrombin signaling through PAR1 activation has previously been implicated in the angiogenic properties of endothelial cells and the formation of new blood vessels (31, 32), although higher concentrations of thrombin can also inhibit angiogenic processes (32). Our data with cleavage-selective PAR1 mutants show that aPC-mediated PAR1 activation is the predominant pathway that supports vascular regeneration in PAD in vivo. This conclusion is furthermore supported by in vitro assays that primarily examine endothelial cell intrinsic processes, including proliferation, migration, and sprouting, and are not dependent on vasodilatation and collateral artery formation, which are relevant for blood flow recovery in vivo. Our data indicate that cleavage at the canonical PAR1 Arg^41^ site makes additional, albeit lesser contributions to neovascularization and specifically supports upregulation of PDGFRB under ischemic conditions. Thus, an additional protease with selectivity for cleaving PAR1 at this position may contribute to the regulation of angiogenesis, besides the delineated EPCR-PAR1 pathway implicating aPC.

The demonstrated role for EPCR-PAR1 signaling in ischemic arterial neovascularization is consistent with prior studies implicating aPC-PAR1 signaling in angiogenesis following brain ischemia (33). aPC stimulates endothelial cell proliferation and new vessel formation in mouse cornea via mechanisms involving eNOS (34), and PAR1 is involved in the sensing of laminar flow and inhibition of eNOS phosphorylation (35). Recombinant aPC protects against ischemic brain damage (36) and ameliorates myocardial (37) or renal (38) ischemia/reperfusion injury. Targeted deletion of TM, critically involved in EPCR-mediated aPC generation, in brain endothelial cells impairs angiogenesis following experimental brain infarction in mice via mechanisms involving NO and VEGF (39). Our analyses extend those previous findings by showing that endothelial EPCR-PAR1 signaling enhances the bioavailability of NO by acting in an autocrine manner and suppressing the expression of hemoglobin, a potent NO scavenger.

NO signaling in endothelial cells reduces the activity of TF (40), and hypoxia-induced HIF1A or VEGF upregulate TF expression (41–43). Our data show that TF is present on the endothelium in peripheral ischemia and contributes to revascularization in the hindlimb model. Functional inhibition of TF reduces angiogenesis, but only in mice with an intact EPCR-PAR1 signaling pathway, indicating that TF is upstream of thrombin and aPC generation or may participate through additional parallel angiogenic signaling pathways for restoration of vessel perfusion in peripheral vascular disease.

Arterial endothelial cells express hemoglobin and use this mechanism to control diffusion and signaling of NO (24, 25), a key signaling mediator involved in the regulation of endothelial cell viability, proliferation, and differentiation. EPCR signaling participates in these processes and has previously been implicated in the differentiation of endothelial stem cells (44) and the regulation of integrin function (45, 46). Increased protein levels of methemoglobin with heme iron oxidized in the ferric state (47) are consistent with the observed increased NO scavenging in EPCR-deficient cells. Methemoglobin is also regulated by hypoxia, and we have found a concordant upregulation of the oxygen-responsive transcription factor NRF2 implicated in fetal hemoglobin expression (27, 28). Our data show that NRF2 is induced during hypoxia in vitro and that endothelial cells exposed to hypoxia internalize EPCR and upregulate NRF2. A function-blocking antibody that neutralizes cell-surface EPCR similarly increased NRF2 expression, supporting a direct role for EPCR cell signaling in NRF2 regulation.

EPCR is present on several cell types involved in new vessel formation (48). Although EPCR is expressed in immune cells under inflammatory conditions (49) and only at low levels on capillary endothelial cells (50), our data implicate EPCR expression by endothelial but not myelomonocytic cells in neovascularization under ischemic conditions. Progenitor cells contribute to endothelial regeneration and angiogenesis (51), and EPCR is expressed on hematopoietic stem cells (52) and identifies vascular stem/progenitor cells with potential for endothelial cell lineage differentiation (44). CD31 was used as a pan–endothelial cell marker but may also have labeled platelets, monocytes, neutrophils, macrophages, and lymphocytes, some of which are known to infiltrate muscle following ischemic injury. On the other hand, the analysis of mice lacking PAR2 and PAR4 strongly suggested that PAR signaling on inflammatory cells or platelets makes no measurable contribution to our findings.

We also show that decreased NO bioavailability or signaling promotes mesenchymal marker expression, myofibroblast accumulation, and fibrosis, all of which are of potential relevance for progression of PAD. Reduced NO bioavailability may cause phenotypic transition of endothelial cells toward myofibroblasts and increased fibrosis, as seen in alveolar epithelial cells after suppression of eNOS with L-NAME (53) or in murine pulmonary endothelium during breast cancer growth (54). In contrast, TGF-β–induced myofibroblast differentiation is attenuated by NO (55) or sGC activation (56). Our data show that hypoxia initiates EPCR internalization, which may explain the lower endothelial EPCR expression and soluble EPCR levels seen in patients with chronic PAD. Whereas EPCR is abundantly expressed on healthy endothelial cells, endothelial dysfunction may enhance proteolytic receptor shedding (57), and significantly higher baseline sEPCR levels have been observed in patients with ST elevation myocardial infarction compared with patients with stable angina pectoris (58) or in diabetic patients with platelet calpain-induced thromboinflammatory PAR1 activation (12).

The presented mechanistic insights into coagulation factor signaling during angiogenesis may help to develop strategies to modulate coagulation signaling for improved postischemic tissue repair without affecting hemostasis in human ischemic PAD.

## Methods

### Experimental animals.

EPCR^fl/fl^ mice were generated from EUCOMM Procr^tm1a(EUCOMM)Wtsi^ embryonic stem cell EPD0480_1_A01 by blastocyst injection and germ line breeding with albino C57BL/6 mice. After conversion to the conditional Procr^tm1c^ allele by a cross with a flippase-expressing line, EPCR^fl/fl^ mice were crossed with either Tie2.Cre (The Jackson Laboratory; B6.Cg-Tg[Tek-cre]12Flv/J; no. 004128) or LysM.Cre (The Jackson Laboratory; Lystm1[cre]Ifo; no.004781) mice. The EPCR and TM genes are in close proximity to the agouti locus in mice. TM^Pro/Pro^ mice were generated in 129/SvPas embryonic stem cells (14, 59). We used TM^Pro/Pro^ mice on a mixed (129/SvPas-C57BL/6) genetic background with a brown fur color or TM^Pro/Pro^ backcrossed to C57BL/6N until a crossover in the fur color locus converted the color from brown to black. This resulted in a difference in lung EPCR expression from high, typical for the 129/SvPas, to low, typical for the C57BL/6 allele. Mice deficient in PAR1 were generated from F2r^tm1aWtsi^ (embryonic stem cell HEPD0904_4_C05; EUCOMM), followed by crossing with an ubiquitously expressed cre deleter (CMV-cre) (60). Offspring of all crosses were screened for expression of the *Nnt* gene that is deleted in C57BL/6J but present in C57BL/6N to assure strain identity. Mouse primer sequence and PCR conditions for genotyping are shown in Supplemental Table 4. Mice with point mutations of PAR1 R41Q or PAR1 R46Q (19) were also on a C57BL/6N background. Mice deficient in PAR2 (PAR2^–/–^) were generated from F2rl1^tm3.1Ruf^ by crossing with CMV-cre (61). Mice deficient in PAR4 (PAR4^–/–^; B6.129S4[FVB]-F2rl3^tm1.1^ ^Cgh^) (62) were on a C57BL/6N genetic background and confirmed to carry the *Nnt* gene expressed by C57BL/6N.

### Mouse model of unilateral hindlimb ischemia.

Unilateral hindlimb ischemia was induced in mice aged 12–14 weeks, as described (63, 64), with minor modifications. Briefly, mice were anesthetized by i.p. injection of ketamine hydrochloride (75 mg/kg body weight; Hameln Pharma Plus) and xylazine (15 mg/kg body weight; Bayer). Anesthesia depth was monitored by observation of the respiratory rate or the toe-pinch reflex test. The right *A*. *saphena* and the right *A*. *femoralis* were ligated, the latter immediately below the inguinal ligament and proximal to the branch point of the *A*. *profunda femoris* followed by the total excision (stripping) of the arterial segment between both ligations to induce ischemia in the calf muscle and angiogenesis and to prevent arterial collateral formation (65, 66). The left, contralateral hindlimb was used as internal control. To examine the role of TF during ischemia, recombinant NAPc2 from Corvas was i.p. injected immediately after surgery and every other day at a dose of 0.5 mg/kg body weight. Phosphate-buffered saline (PBS) was injected at the same volume in the control animals. In all experiments, only male mice were used to avoid estrogen cycle dependent variability of blood flow recovery after hindlimb ischemia. At the end of the observation period of 28 days, deeply anesthetized mice were killed by cervical dislocation, and tissues were processed as described below.

### LDPI.

Immediately before and after surgical induction of hindlimb ischemia, as well as during follow-up examinations on days 7, 14, 21, and 28, mice anesthetized with isoflurane (1.5%–2%) were placed on 37°C heated pads, and blood perfusion in both feet was determined by LDPI (PIM III, Perimed). Duplicate measurements were averaged for quantification of perfusion. Values were calculated per region-of-interest (footpad) and are expressed as percentage of blood flow compared with the contralateral, nonischemic site used as internal control.

### Immunofluorescence analysis and confocal microscopy.

Capillary density in the gastrocnemius muscle was assessed 28 days after induction of unilateral hindlimb ischemia on 5 μm–thick, acetone-fixed frozen sections (for 10 minutes at –20°C) stained with lycopersicon esculentum lectin or monoclonal rat antibodies against murine CD31 (Pecam1; Santa Cruz Biotechnology; clone MEC13.3; sc-18916; dilution, 1:50) to label immature, angiogenic endothelial cells (67), as well as antibodies against CD41 (Itga2b, Biozol; 11-763-C100; dilution, 1:100) to label platelets, eNOS (D9A5L; Cell Signaling Technology; 32027; dilution, 1:100), EPCR (R&D Systems; AF2245; dilution, 1:100), fibrinogen (Abcam; clone EPR18145-84; ab189490; dilution, 1:100), HBA (LifeSpan BioSciences; LS-B10549 1:100), PDGFRB (Abcam; ab32570; dilution, 1:100), and α-smooth muscle actin (α-SMA; clone 1A4; Sigma-Aldrich; A2547; dilution, 1:50) to label smooth muscle cells or TF (R1073; dilution, 1:100) (68). Cell nuclei were counterstained with DAPI (Roth; 6335.1; dilution, 1:1,000). Immunofluorescent images were collected using a Leica LSM710 confocal microscope or high-resolution, confocal-quality Keyence BZ-X800 microscope, and they were analyzed with Leica software (LAS X) or Keyence analysis software. Interstitial collagen was detected 28 days after induction of unilateral hindlimb ischemia using picrosirius red staining, followed by microscopy under polarized light. The number of immunopositive cells per single muscle fiber was manually counted on 4 random microscope fields per section (×200 magnification), and results were averaged per mouse.

### Flow cytometry analysis and DAF-2 DA staining.

Isolated lower limb muscle was minced and digested with collagenase type IV (Worthington; final concentration, 2 mg/mL) and 2 mM CaCl_2_ in PBS at 37°C for 45 minutes. Cells were fixed using 0.1% PFA for 10 minutes at room temperature (RT), followed by permeabilization using 0.1% Triton X-100 (Thermo Fisher Scientific) for 5 minutes at RT. Unspecific binding was blocked using FcR receptor blocking reagent (Miltenyi Biotec; 130-092-575), followed by incubation with allophycocyanin-labeled primary antibodies against CD31 (CD31-APC; clone MEC13.3; BioLegend; 102510; 1:100). To visualize NO, 1 × 10^6^ unfixed cells were incubated with fluorescent NO probe 1.5 μM DAF-2 DA (Abcam; ab146631) at 37°C for 35 minutes, based on preliminary dose-finding studies and as published previously (69). Cells were washed and immediately analyzed using flow cytometry (BD Biosciences FACSCanto II). Forward and side scatter gates were set to exclude debris and cellular aggregates. Unstained cells were used as a negative controls.

### Primary murine endothelial cell isolation.

Mouse primary pulmonary endothelial cells (mPECs) were isolated, as described (70). Briefly, lungs were excised, washed, diced, and incubated in digestion buffer (1.5 mg/mL of collagenase A in PBS). Lung lysates were first negatively selected using magnetic CD45-labeled MicroBeads (Miltenyi Biotec; 130-052-301) to remove leucocytes followed by CD31^+^ selection (Miltenyi Biotec; 130-097-418) on magnetic separation LS columns (Miltenyi Biotec; 130-042-401), and they were directly subjected to lysis and mRNA or protein isolation, or to functional analysis using the spheroid angiogenesis assay.

### Human microvascular endothelial cells.

Human cardiac microvascular endothelial cells (HCMECs) were cultivated, as suggested by the supplier (PromoCell; C-12286). At 80%–90% confluency, cells were treated with DETA-NO (100 μM; Abcam), PTIO (2,-phenyl-4, 4, 5, 5,-tetramethylimidazoline-1-oxyl 3-oxide; 100 μM; Sigma-Aldrich), or ODQ (50 μM; Tocris) for 4 days. In some experiments, cells were cultivated in MV2 medium containing 5% human serum and pretreated with 50 nM recombinant NAPc2, or they were cultivated in MV2 medium containing 5% human serum and treated with 20 μg/mL of EPCR blocking antibody 1496 (6). To recapitulate hypoxia, HCMECs were cultivated under 1% oxygen for 24 hours (Thermo Fisher Scientific; HERACell Vios160i). All human primary cells were used between passage 4 and passage 6.

### Angiogenesis assays.

The mouse aortic ring assay was performed, as described previously (71). Briefly, murine aortas were cut into 2 mm pieces, embedded in phenol-free matrigel (Corning; 356237), and cultivated for 4 days in Endothelial Cell Growth Medium MV2 kit (PromoCell; C-22121). To study endothelial sprout formation, mPECs or HCMECs were subjected to the spheroid assay, as published (63). A total of 3.2 × 10^4^ of mPECs or HCMECs were resuspended in 10 mL MV2 containing 20% methylcellulose solution and incubated overnight. The next day, spheroids were collected and embedded in collagen and incubated for 24 hours at 37°C. Pictures of 10 spheroids at random phase contrast microscopy fields were taken and analyzed by image analysis software (ImageJ; NIH). In some experiments, aortic rings or spheroids were incubated with TR41 and TR47 peptides (10) (20 μM; PepMic) and their respective controls — scrambled (scr) TR41 and TR47 (20 μM; PepMic) — or with DETA-NO (100 μM; Abcam).

### qPCR.

Total RNA was isolated using Trizol reagent (Ambion; 15-596-018). In total, 1 μg of RNA was treated with RQ1 RNase-Free DNase I to eliminate genomic DNA (Promega; M6101) and reversed transcribed into cDNA using iScript cDNA Synthesis Kit (Bio-Rad; 1708890), followed by qPCR. All qPCR data are normalized to HPRT1 (hypoxanthine-guanine phosphoribosyltransferase; Eurofins) and are reported as fold change versus control, as specified in the text. Primer sequences and PCR conditions are shown in Supplemental Table 5 for mice and Supplemental Table 6 for humans.

### Protein isolation and Western blot analysis.

mPECs were lysed in RIPA buffer (Cell Signaling Technology) containing 1 mM PMSF (phenylmethanesulfonyl fluoride; Cell Signaling Technology). Equal amounts of protein were fractionated by SDS polyacrylamide gel electrophoresis together with molecular weight standards and transferred to nitrocellulose membranes (Protran, Whatman). Membranes were blocked in 5% BSA (in TBS/0.1% Tween-20), followed by incubation with polyclonal antibodies against EPCR (R&D Systems; AF2245; dilution, 1:1,000), eNOS (Cell Signaling Technology; D9A5L; #32027; dilution, 1:1,000), HBA (LifeSpan Biosciences; LS-B10549; dilution, 1:1,000), HIF1α (Abcam; ab2185; dilution, 1:1,000), methemoglobin (HBG2; Invitrogen; PA5-97822; dilution, 1:1,1000), or NRF2 (clone A-10; Santa Cruz Biotechnology; sc-365949; dilution, 1:1,000). Antibodies against β-actin (ACTB; Abcam; ab8226; dilution, 1:1,000) or α-actinin (ACTNA; Cell Signaling Technology; 3134; dilution, 1:1,000) were used to assess total protein loading. Protein bands were visualized using horseradish peroxidase–conjugated secondary antibodies (Amersham Biosciences) and detected with SuperSignal West Pico Substrate (Pierce).

### Analyses in patients with ischemic PAD.

Plasma was obtained from patients (*n* = 19; 47.4% male; mean age, 67.4 ± 1.8 years) with symptomatic PAD (classified as Rutherford stage 1–3). Patients were recruited via the Department of Cardiology, Cardiology III, University Medical Center Mainz, Germany. Nineteen age- and sex-matched persons (*n* = 19; 47.4% male; mean age, 66.9 ± 1.6 years) with similar cardiovascular risk factors but no diagnosis of PAD were used as controls and recruited via the Gutenberg Health Study, Department of Cardiology, Section Preventive Cardiology, University Medical Center Mainz, Germany. Plasma-soluble EPCR was determined using an enzyme-linked immunoassay (MBS703733; MyBioSource; minimum detectable dose of less than 1.95 ng/mL). Production of NO was examined by determining plasma nitrite/nitrate levels using Griess Reagent System (Promega; G2930; detection limit of 125 pmol) (72). Vascular specimens were obtained from symptomatic patients with lower extremity ischemia (*n* = 4) undergoing elective surgery at the University Hospital Alexandroupolis, Department of Vascular Surgery, Alexandroupolis, Greece. Skeletal muscle tissue from the lower extremities of patients with nonischemic disease was used as control (*n* = 4). Tissue specimens were immediately processed for paraffin embedding and cut into 5 μm–thick serial sections. Expression of endothelial specific expression (CD31; Dianova; DIA-310; dilution, 1:50) of HBA (Abcam; ab191183; dilution, 1: 250), eNOS (Cell Signaling Technology; 32027; clone D9A5L; dilution, 1:100), and EPCR (R&D Systems; AF2245; dilution, 1:40) was examined. Patients with active cancer or with systemic inflammatory or autoimmune disease were excluded from all analyses.

### Data availability.

The data underlying this article are available in the article and in its supplemental material online.

### Statistics.

Quantitative data are reported as mean ± SEM. Normal distribution was examined using the D’Agostino & Pearson omnibus normality test. For comparison of 2 groups and normal distribution, 2-tailed Student’s *t* test was performed. More than 2 groups were compared with 1-way or 2-way ANOVA, if more than 1 time point was compared, followed by multiple-comparison tests (Sidak’s or Bonferroni’s), as recommended by the statistical program. Nonparametric tests were used if normal distribution was not present. Statistical differences were assumed if *P* reached a value less than 0.05. All analyses were performed using GraphPad PRISM data analysis software (version 9.0.1; GraphPad Prism Software Inc.).

### Study approval.

All animal care and experimental procedures were approved by the State Board of Animal Welfare (approval no. G 16-1-049) in accordance with the *Guide for the Care and Use of Laboratory Animals* (National Academies Press, 2011). All human study protocols complied with the declaration of Helsinki and were approved by the IRB in Mainz (approval no. 2019-14205-Klinische Forschung). All participants were informed and gave their signed consent prior to inclusion in the study.

## Author contributions

Research study design was contributed by MLB, WR, and KS. Conducting experiments was contributed by MLB, RG, SG, JK, and JO. Original draft writing, reviewing, and editing were contributed by MLB, WR, and KS. Provision of resources was contributed by SR, GSG, HMS, SK, TM, JHG, PW, and CEK. With the exceptions of first and senior authors, the order of authors reflects the experimental contributions as well as timing of involvement with the project.

## Supplementary Material

Supplemental data

## Version 1 06/14/2022

In-Press Preview

## Version 2 07/22/2022

Electronic publication
