# Supplementary material for: EPCR-PAR1 biased signaling regulates perfusion recovery and neovascularization in peripheral ischemia
Source: JCI Insight. 2022 Jul 22;7(14):e157701. doi: 10.1172/jci.insight.157701 (PMC9431695; doi:10.1172/jci.insight.157701)
Supplement: Supplemental data [file jciinsight-7-157701-s104.pdf]

ONLINE SUPPLEMENTARY MATERIAL

EPCR–PAR1 biased signaling regulates perfusion recovery and neovascularization in peripheral ischemia

Magdalena L. Bochenek,[1,2] Rajinikanth Gogiraju,[2] Stefanie Großmann,[2] Janina Krug,[2] Jennifer Orth,[2] Sabine Reyda,[1] George S. Georgiadis,[3] Henri M. Spronk,[4] Stavros Konstantinides,[1,5] Thomas Münzel,[2,6] John H. Griffin,[7] Philipp Wild,[1,2,6] Christine Espinola-Klein,[8] Wolfram Ruf[1,6,9]* and Katrin Schäfer[2,6]*

* These authors share the senior authorship.

Supplemental Figures

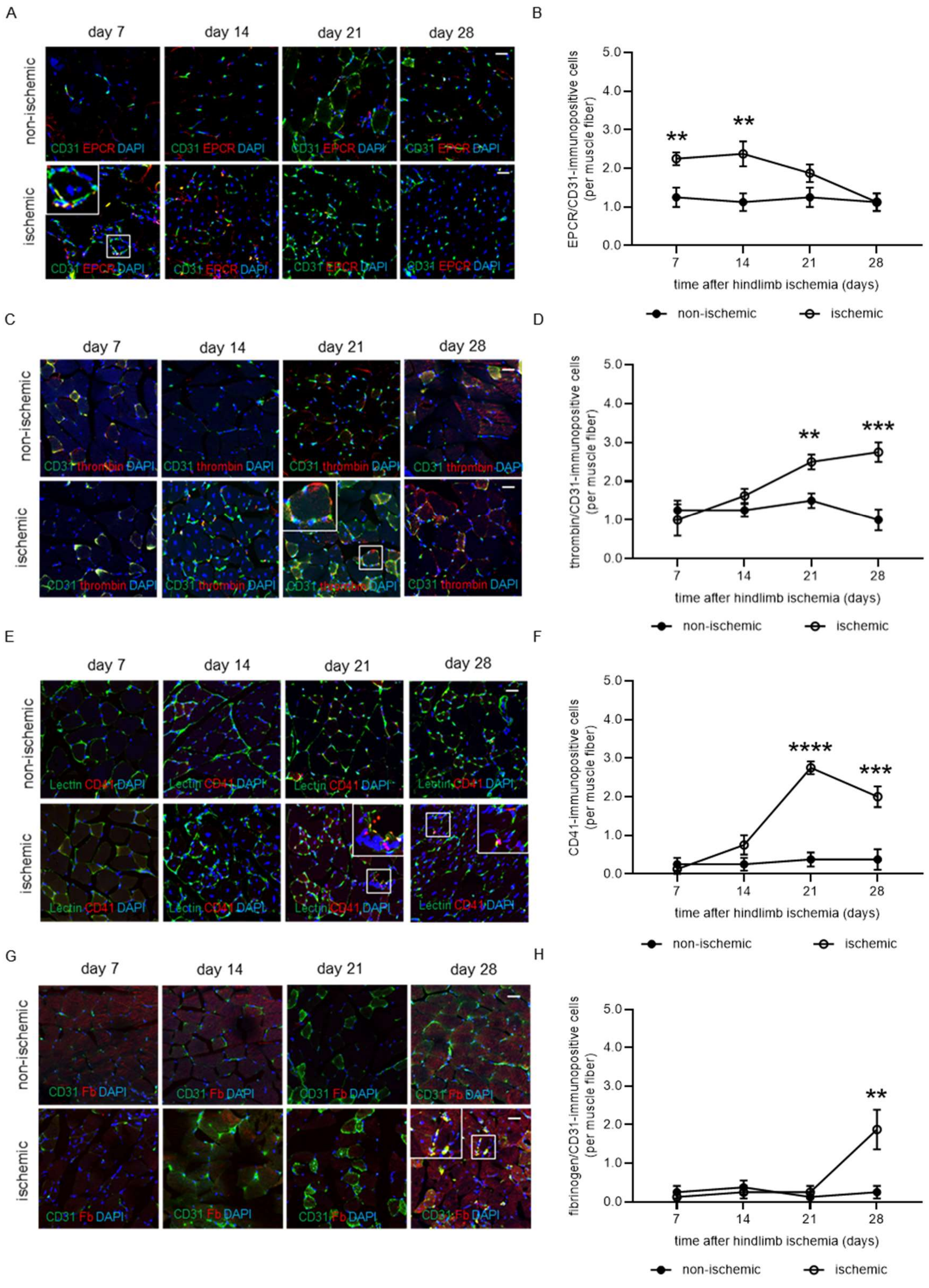

Supplemental Figure 1. Time curve analysis of EPCR, thrombin and fibrinogen expression and the presence of platelets in the murine hindlimb ischemia model. (**A**) Representative confocal microscopy images and (**B**) quantification of cryo-preserved cross-sections of non-ischemic and ischemic hindlimbs in C57BL/6N mice at day 7, day 14, day 21 and day 28 after surgery and immunostaining for CD31 (green) and EPCR (red); n=4

biological replicates per time point. (**C**) Representative confocal microscopy images and (**D**) quantification of cryo-preserved cross-sections of non-ischemic and ischemic hindlimbs in C57BL/6N mice at day 7, day 14, day 21 and day 28 after surgery and immuno-stained for CD31 (green) and thrombin (red); n=4 biological replicates per time point. (**E**) Representative confocal microscopy images and (**F**) quantification of cryo-preserved cross-sections of non-ischemic and ischemic hindlimbs in C57BL/6N mice at day 7, day 14, day 21 and day 28 after surgery and stained for endothelial lectin (green) and CD41 to label platelets (red); n=4 biological replicates per time point. (**G**) Representative confocal microscopy images and (**H**) quantification of cryo-preserved cross-sections of non-ischemic and ischemic hindlimbs in C57BL/6N mice at day 7, day 14, day 21 and day 28 after surgery stained for CD31 (green) and fibrinogen (Fb; red); n=4 biological replicates per time point. **p<0.01, ***p<0.001 and ****p<0.0001 vs. findings in the contralateral, non-ischemic leg at the same time point. Data were analyzed using multiple t tests. Scale bars = 10 µm.

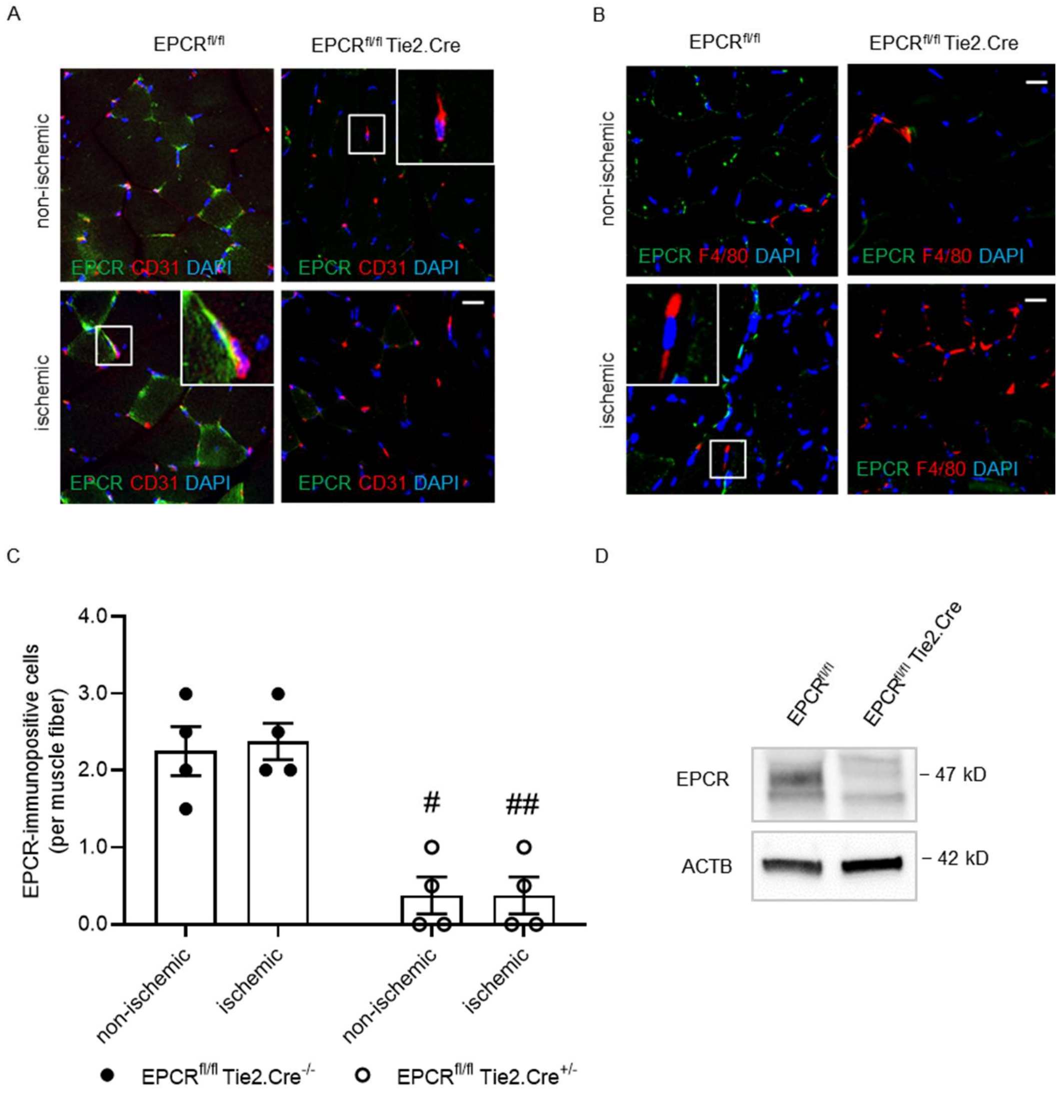

Supplemental Figure 2. Endothelial EPCR expression in EPCR$^{fl/fl}$ Tie2.Cre mice. (**A**, **B**) Representative confocal microscopy images and (**C**) quantification of cryo-preserved cross-sections of non-ischemic and ischemic hindlimbs of EPCR$^{fl/fl}$ Tie2.Cre and EPCR$^{fl/fl}$ littermate controls stained with antibodies to (**A**) EPCR (green) and CD31 (red) or (**B**) EPCR (green) and F4/80 (red). DAPI-positive cell nuclei appear blue. Scale bars represent 10 µm; n=4 biological replicates. #p<0.05 and ##p<0.01 vs. EPCR$^{fl/fl}$ control littermate mice; Two-way ANOVA, Sidak's multiple comparisons test. (**D**) Representative western blots for EPCR protein in murine endothelial cells isolated from lungs of EPCR$^{fl/fl}$ Tie2.Cre mice and littermate controls; n=3 biological replicates.

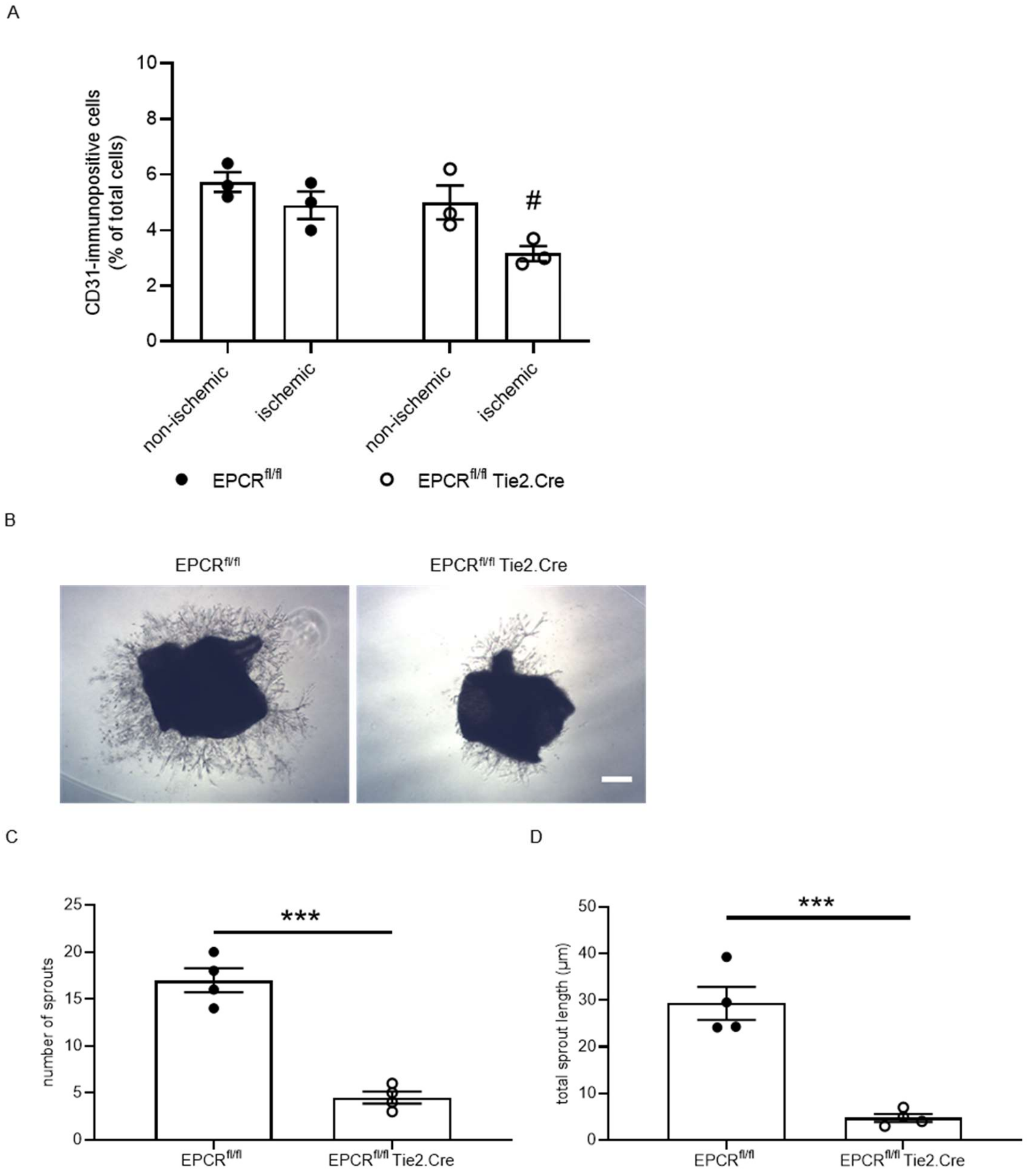

Supplemental Figure 3. EPCR-dependent endothelial sprouting. (**A**) Results of the flow cytometry quantitative analysis of CD31-positive cells in non-ischemic and ischemic hindlimbs in EPCR$^{fl/fl}$ Tie2.Cre mice and EPCR$^{fl/fl}$ littermate controls; n=3 biological replicates. #$p<0.05$ vs. EPCR$^{fl/fl}$ littermates; Two-Way ANOVA, Sidak's multiple comparisons test. (**B**) Representative brightfield microscopy images of aortic rings isolated from EPCR$^{fl/fl}$ Tie2.Cre and EPCR$^{fl/fl}$ littermate controls. Scale bar = 10 µm. (**C**) Quantification of number of sprouts per aortic ring and (**D**) total length of sprouts; n=4 biological replicates. ***$p<0.001$; Student's t-test.

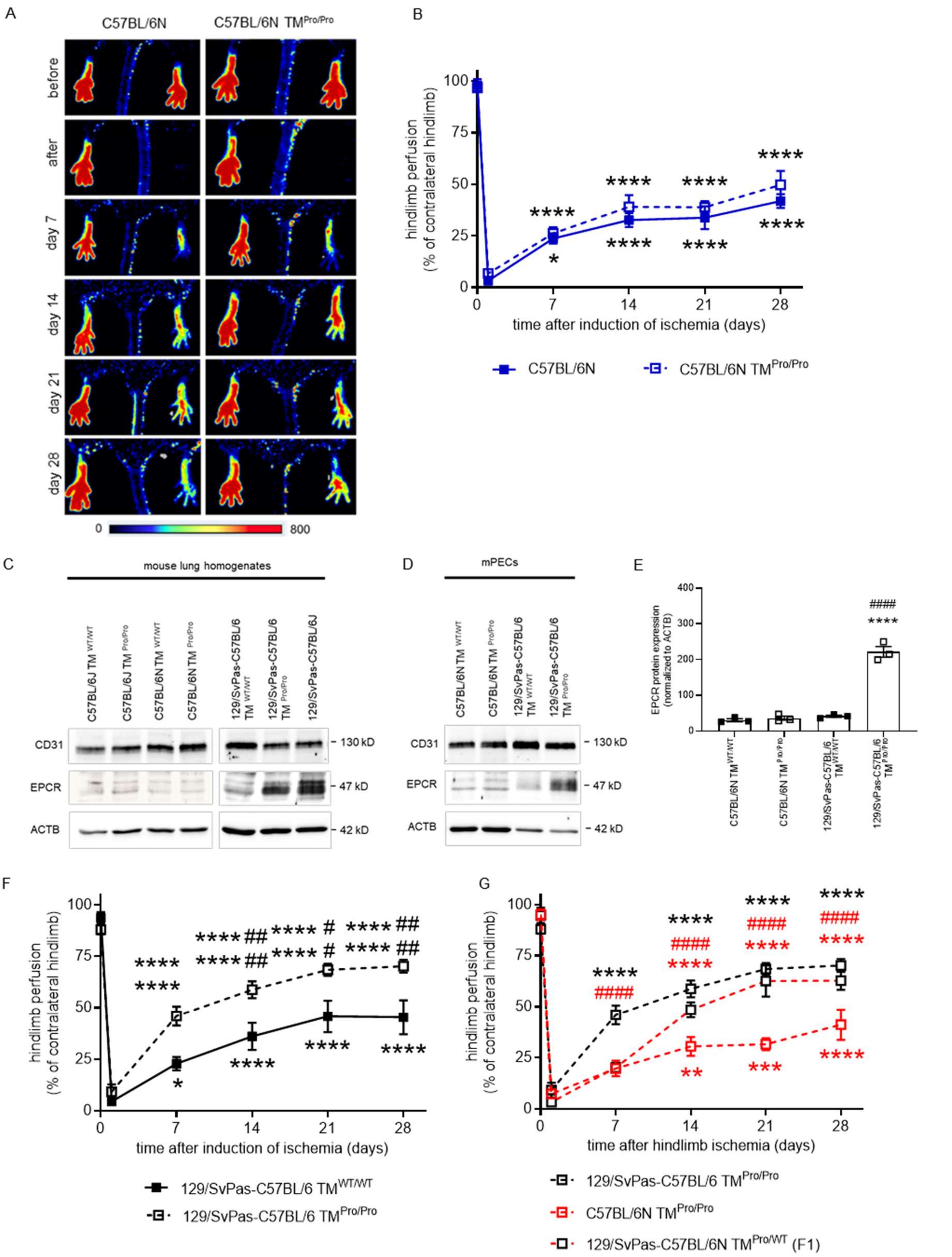

Supplemental Figure 4. Improved hindlimb reperfusion and neovascularization in the presence of increased EPCR expression. (**A**) Representative laser Doppler perfusion images in C57BL/6N TM$^{Pro/Pro}$ mice (n=10) and C57BL/6N controls (n=14) immediately before and after as well as on day 7, day 14, day 21 and day 28 after induction of hindlimb ischemia. (**B**) Quantitative analysis of the laser signal (expressed as % of the contralateral, non-ischemic site). *p<0.05 and ****p<0.0001 vs. values immediately after surgery; Two-Way ANOVA, Sidak's multiple comparisons test. Non-significant p values are not shown. (**C**) Western blot of mouse lung homogenates and (**D**) of mouse primary endothelial cells (mPECs) isolated

from their lungs. (**E**) Quantification showing EPCR expression normalized to ACTB. ****$p<0.0001$ vs. control littermate mice; ####$p<0.001$ vs. C57BL/6N $TM^{WT/WT}$ mice using Student's t-test; n=3 biological replicates. (**F**) Quantitative analysis of hindlimb reperfusion (expressed as % of the contralateral, non-ischemic site) in 129/SvPas-C57BL6 $TM^{Pro/Pro}$ mice (n=16) and 129/SvPas-C57BL6 $TM^{WT/WT}$ littermate controls (n=9) immediately before and after as well as on day 7, day 14, day 21 and day 28 after induction of hindlimb ischemia. *$p<0.05$ and ****$p<0.0001$ vs. values immediately after surgery; #$p<0.05$ and ##$p<0.01$ vs. 129/SvPas-C57BL6 $TM^{WT/WT}$ controls at the same time point; Two-Way ANOVA, Sidak's multiple comparisons test. Non-significant p values are not shown. (**G**) Quantitative analysis of the laser signal (expressed as % of the contralateral, non-ischemic site) in 129/SvPas-C57BL6 $TM^{Pro/Pro}$ (n=12), C57BL6 $TM^{Pro/Pro}$ (n=9), and F1 progeny of a cross of 129/SvPas with C57BL6 $TM^{Pro/Pro}$ mice (n=5); **$p<0.01$, ***$p<0.001$ and ****$p<0.0001$ vs. values immediately after surgery; ####$p<0.0001$ vs. C57BL/6N $TM^{Pro/Pro}$ at the same time point; Two-Way ANOVA, Sidak's multiple comparisons test.

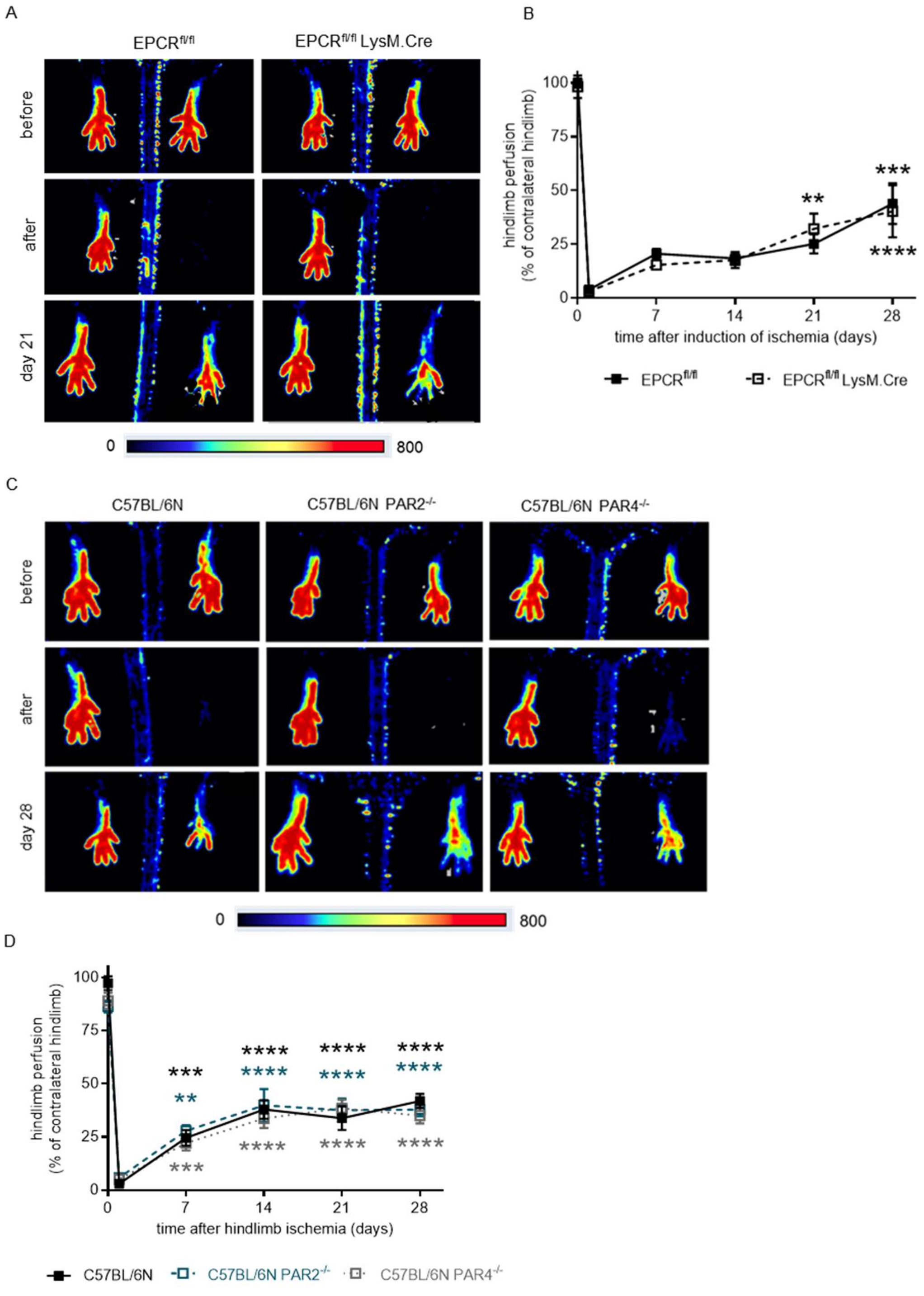

Supplemental Figure 5. Reperfusion following ischemia in mice lacking EPCR in LysM.Cre expressing cells or mice with global PAR2 and PAR4 deletion. (**A**) Representative laser Doppler perfusion images of EPCR$^{fl/fl}$ LysM.Cre mice (n=6) and EPCR$^{fl/fl}$ littermate controls (n=7) immediately before and after and on day 28 after hindlimb ischemia. (**B**) Quantification of the laser signal (as % of the contralateral, non-ischemic site). **p<0.01, ***p<0.001 and ****p<0.0001 vs. values immediately after surgery; Two-Way ANOVA, Sidak's multiple comparisons test. (**C**) Representative laser Doppler perfusion images and (**D**) quantification in C57BL/6N control mice (n=11), C57BL/6N PAR2$^{-/-}$ (n=10) and C57BL/6N PAR4$^{-/-}$ mice (n=8). **p<0.01, ***p<0.001 and ****p<0.0001 vs. values immediately after surgery; Two-Way ANOVA, Sidak's multiple comparisons test.

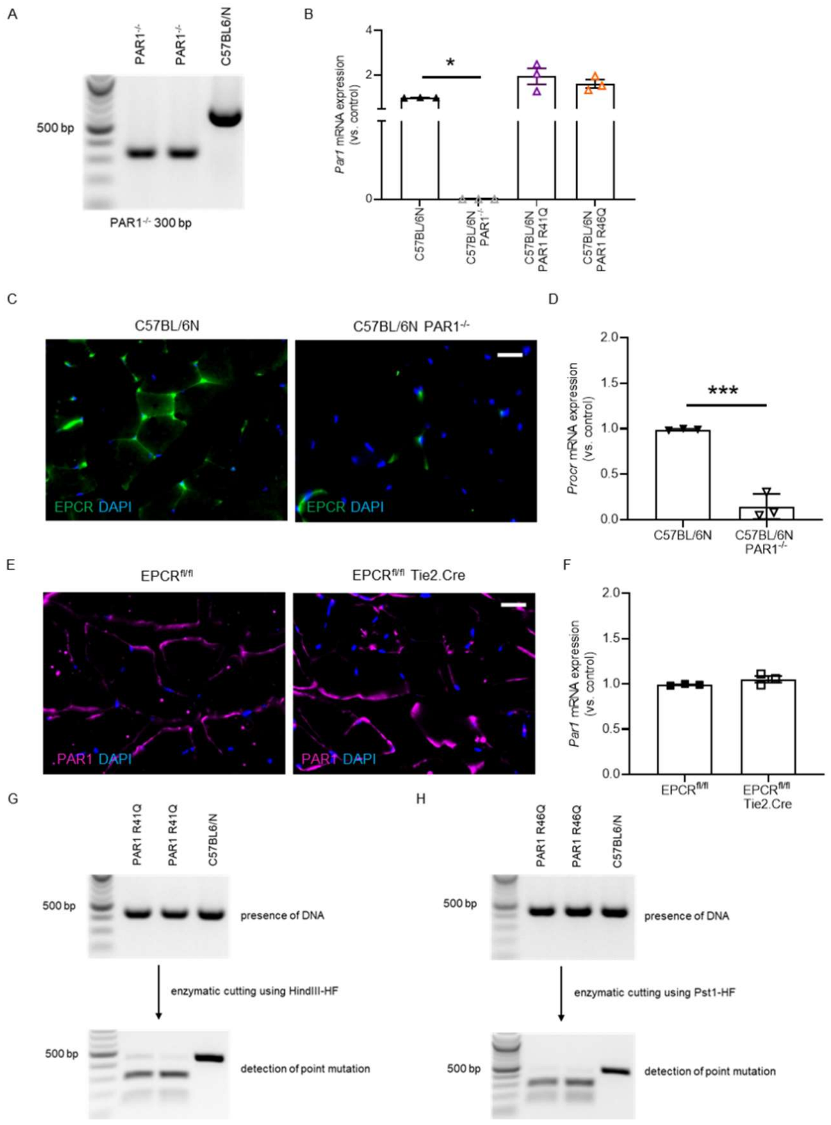

Supplemental Figure 6. Regulation of EPCR and PAR1 expression in PAR1 knockout and PAR1 mutant mice. (**A**) Representative images of agarose gels for PAR1 expression in mouse ear biopsies to confirm PAR1 gene deletion. (**B**) Quantitative polymerase chain reaction analysis of PAR1 expression in primary endothelial cells isolated from C57BL/6N PAR1$^{-/-}$, C57BL/6N PAR1 R41Q, C57BL/6N PAR1 R46Q and C57BL/6N control mice. *$p<0.05$ vs. C57BL/6N control mice; One-Way ANOVA, Dunett's multiple comparisons test; n=3 biological replicates. (**C**) Representative immunofluorescent high-resolution images of EPCR expression (green) in C57BL/6N PAR1$^{-/-}$ and C57BL/6N control mice; n=4 biological replicates. Scale bar represents 10 µm. (**D**) QPCR analysis of EPCR expression in C57BL/6N PAR1$^{-/-}$ and C57BL/6N control mice; n=3 biological replicates. ***$p<0.001$ vs. control mice. (**E**) Representative immunofluorescent high-resolution images of PAR1 expression (purple) in EPCR$^{fl/fl}$ Tie2.Cre mice and their littermate controls; n=4 biological replicates. Scale bar represents 10 µm. (**F**) QPCR analysis of PAR1 expression in EPCR$^{fl/fl}$ Tie2.Cre mice and their littermate controls; n=3 biological replicates. ***$p<0.001$ using Student's t-test. Representative images of agarose gels for (**G**) PAR1 R41Q and (**H**) PAR1 R46Q mice for the presence of DNA and enzymatic reaction using HindIII (PAR1 R41Q) or Pst1 (PAR1 R46Q) to confirm the point mutations, respectively, in mouse ear biopsies.

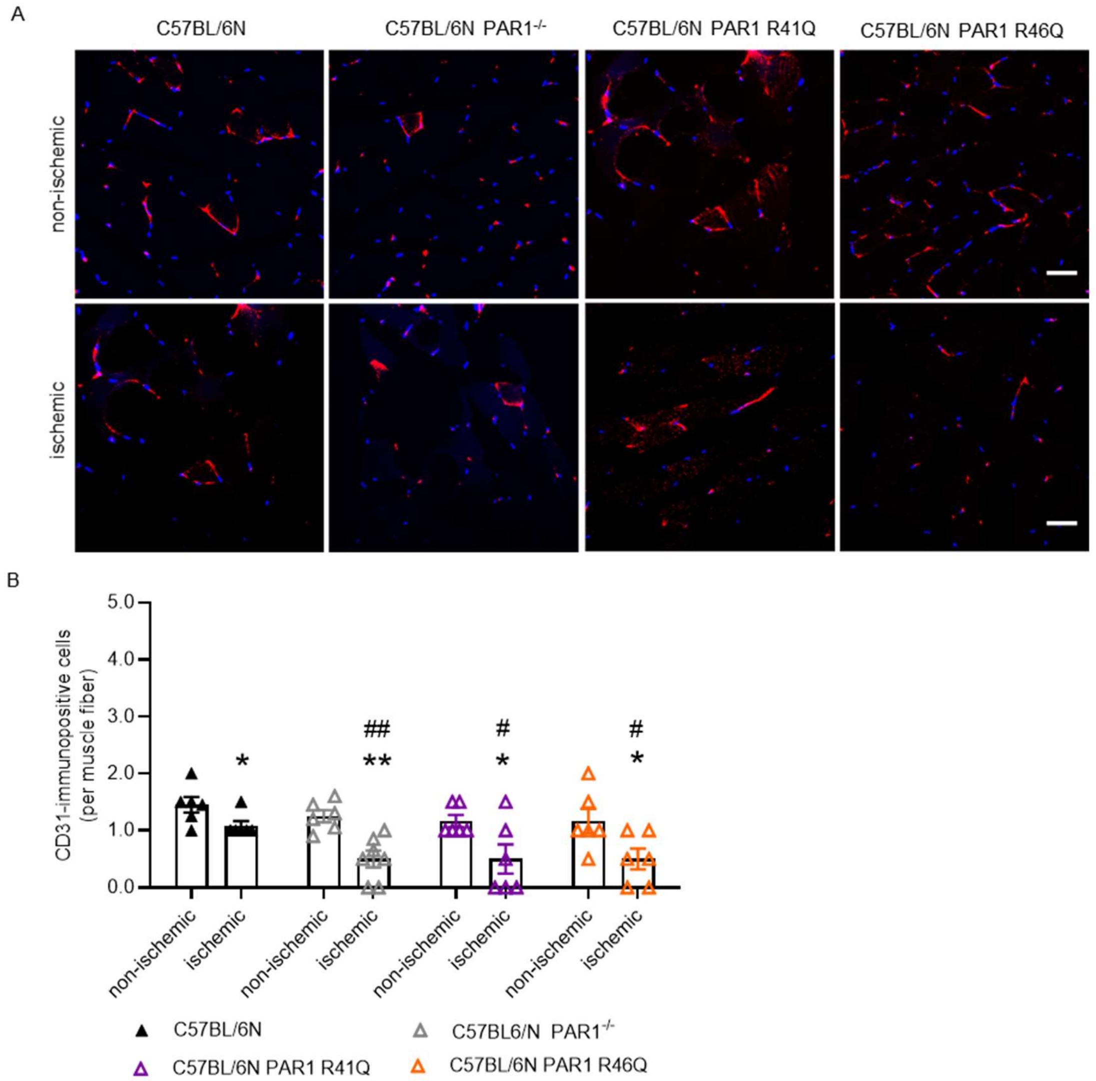

Supplemental Figure 7. Neovascularization in PAR1 mutant mice. (**A**) Representative immunofluorescent confocal images and (**B**) quantification analysis after visualization of CD31-immunopositive cells (red) in non- and ischemic hindlimbs of PAR1$^{-/-}$, PAR1 R41Q, PAR1 R46Q and wild-type C57BL/6N control mice; n=5 biological replicates. DAPI positive cells nuclei appear blue. Scale bars represent 10 µm. *p<0.05 and **p<0.01 vs. non-ischemic control muscle; #p<0.05 and ##p<0.01 vs. ischemic muscle of C57BL/6N mice; Two-Way ANOVA, Sidak's multiple comparisons test.

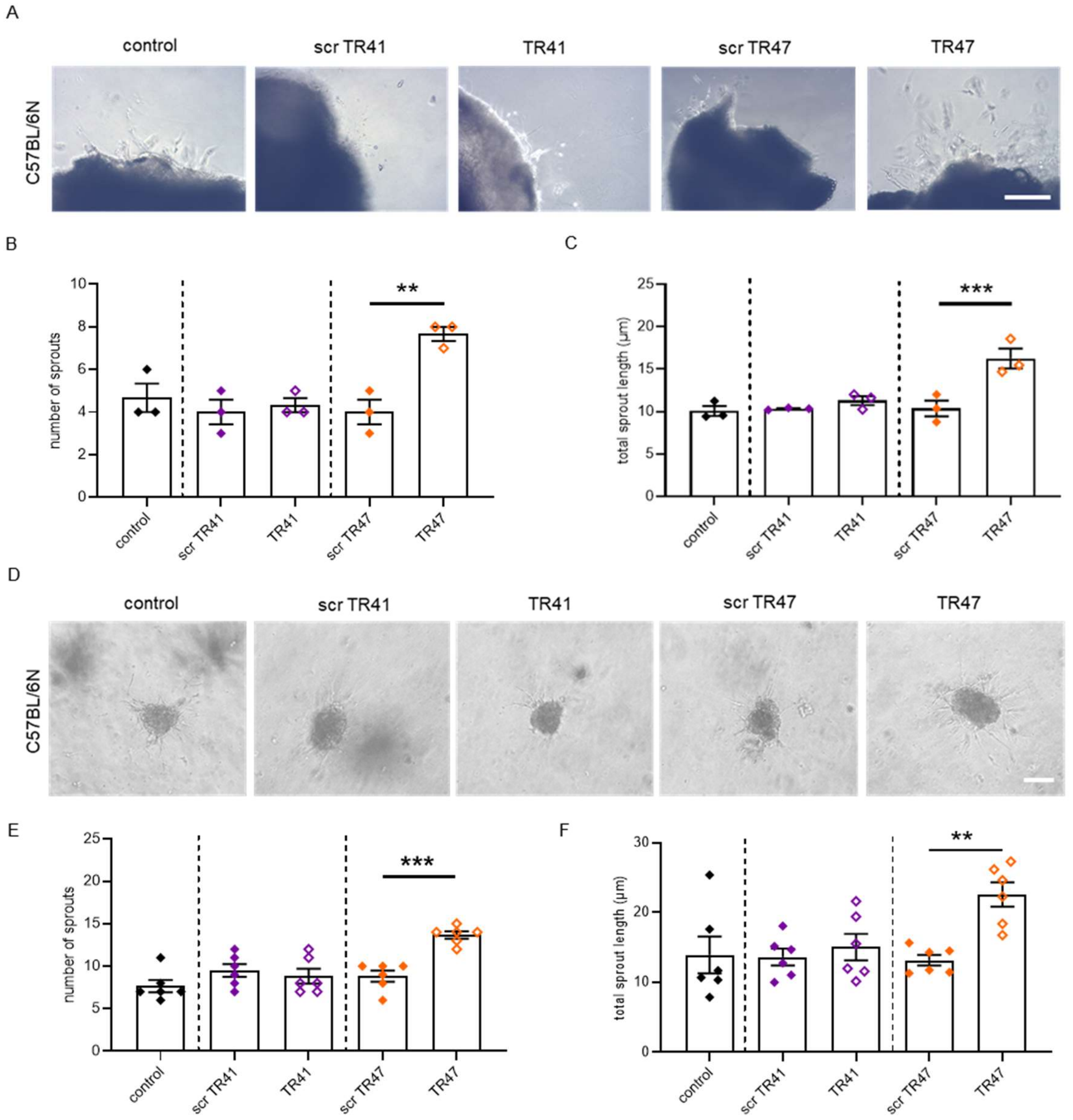

Supplemental Figure 8. PAR1 biased signaling and ex vivo and in vitro analysis of sprouting angiogenesis. (**A**) Representative brightfield images of aortic rings isolated from C57BL/6N mice, untreated (control) or treated with TR41 and TR47 PAR1-based peptides mimicking activation by thrombin or aPC cleavage, respectively, or scrambled (scr) control peptides. Scale bar represents 10 µm. (**B**) Quantitative analysis of the number of sprouts or (**C**) the total sprout length; n=3 biological replicates. ***p<0.001 vs. scr control peptide; One-Way ANOVA, Sidak's multiple comparisons test. (**D**) Representative brightfield images of mPECs isolated from C57BL/6N mice and subjected to the spheroid assay, untreated (control) or treated with TR41 and TR47 PAR1-based peptides or scr control peptides. Scale bar represents 10 µm. (**E**) Quantification of sprouts number or (**F**) sprout length; n=3 biological replicates á 2 experimental repeats. **p<0.01 and ***p<0.001 vs. scr control peptides; One-Way ANOVA, Sidak's multiple comparisons test.

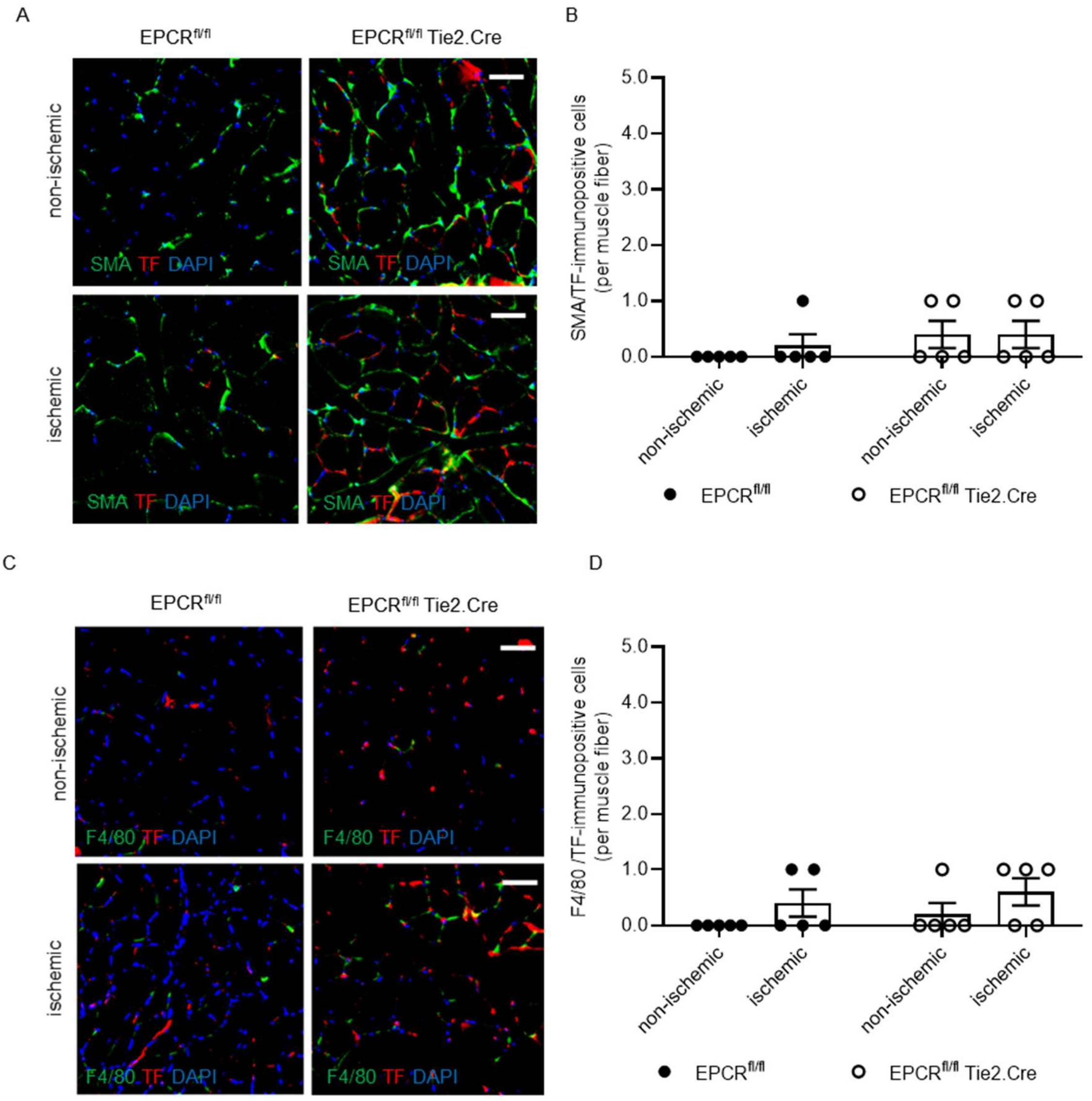

Supplemental Figure 9. Cell type-specific tissue factor expression upon EPCR deletion. (**A**) Representative high-resolution images and (**B**) quantitative analysis after staining of ischemic and non-ischemic lower hindlimb muscles of EPCR$^{fl/fl}$ Tie2.Cre mice and EPCR$^{fl/fl}$ littermate controls with SMA (green) and TF (red); n=5 biological replicates. DAPI-positive cell nuclei appear blue. Scale bars represent 10 µm. (**C**) Representative high-resolution images and (**D**) quantitative analysis after staining of ischemic and non-ischemic lower hindlimb muscles of EPCR$^{fl/fl}$ Tie2.Cre mice and EPCR$^{fl/fl}$ littermate controls with F4/80 (green) and TF (red); n=5 biological replicates. DAPI-positive cell nuclei appear blue. Scale bars represent 10 µm. Two-Way ANOVA, Sidak's multiple comparisons test.

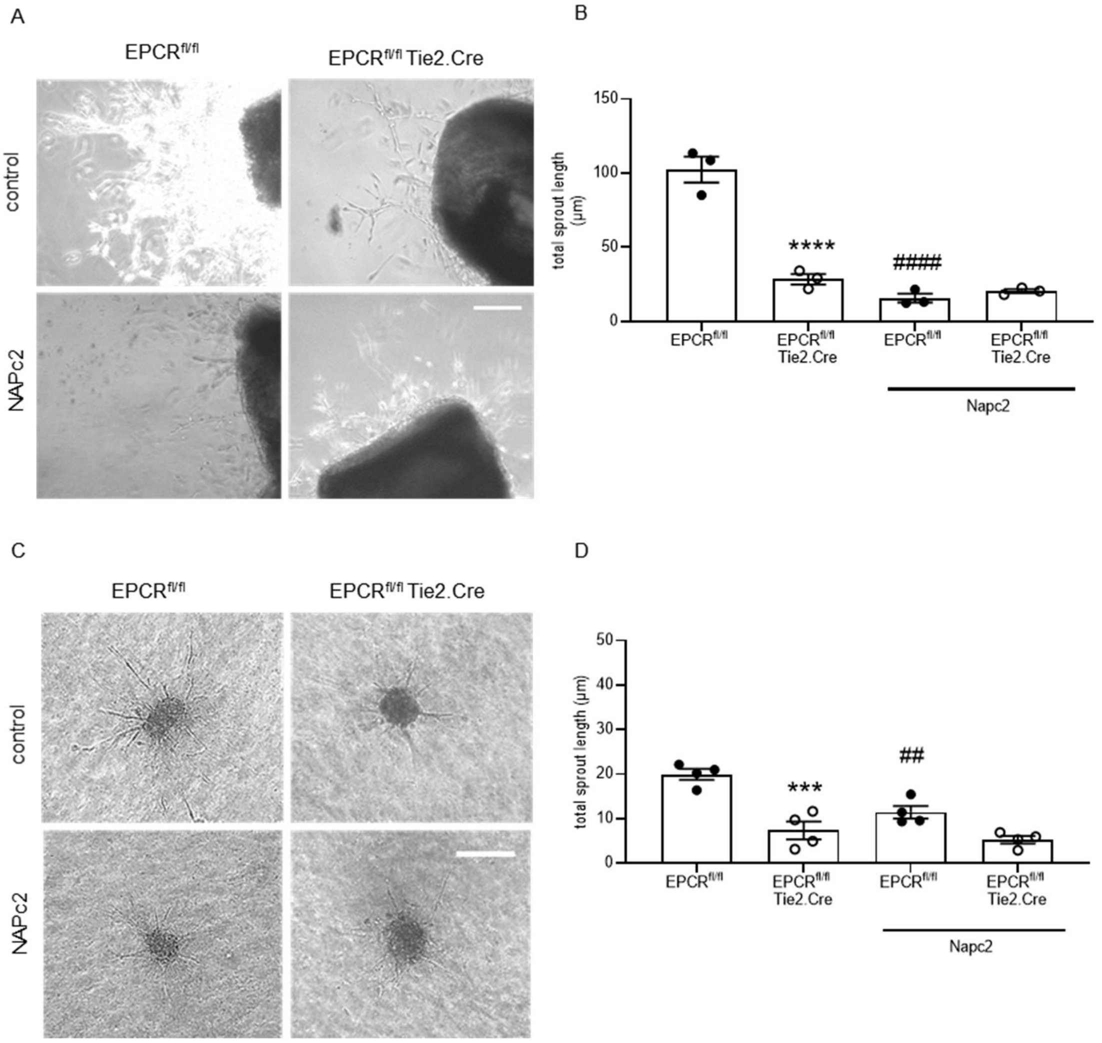

Supplemental Figure 10. Sprouting angiogenesis in EPCR$^{fl/fl}$ Tie2.Cre mice upon inhibition of tissue factor. (**A**) Representative brightfield images and (**B**) quantitative analysis of total sprout length of aortic rings isolated from EPCR$^{fl/fl}$ Tie2.Cre and EPCR$^{fl/fl}$ littermate controls with and without NAPC2. Scale bar represents 10 µm. ****$p<0.0001$ vs. EPCR$^{fl/fl}$ mice; ####$p<0.0001$ for NAPc2 vs. untreated cells isolated from mice with the same genotype using Two-way ANOVA, Sidak's multiple comparisons test; n=3 biological replicates. Non-significant p values are not shown. (**C**) Representative brightfield images and (**D**) quantitative analysis of CD31-positive endothelial cells isolated from EPCR$^{fl/fl}$ Tie2.Cre and EPCR$^{fl/fl}$ littermate controls subjected to the spheroid assay with and without NAPc2; n=4 biological replicates. Scale bar represents 10 µm. ***$p<0.001$ vs. EPCR$^{fl/fl}$ mice; ##$p<0.01$ for NAPc2 vs. untreated cells isolated from mice with the same genotype using Two-way ANOVA, Sidak's multiple comparisons test.

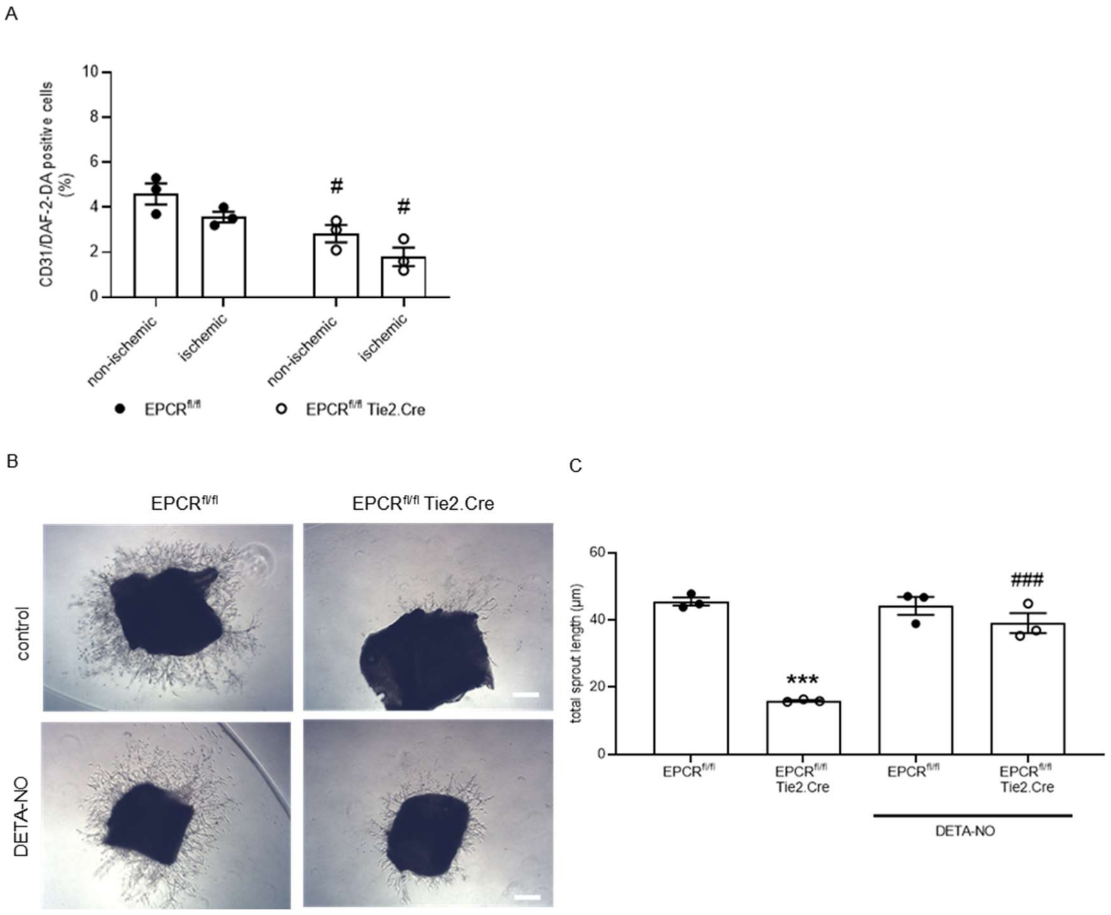

Supplemental Figure 11. EPCR-dependent effects on nitric oxide. (**A**) Flow cytometry analysis of CD31/DAF-2-DA-double positive cells isolated from mouse hindlimb muscles at day 28 after ischemia; n=3 biological replicates; Two-Way ANOVA, Sidak's multiple comparisons test. #p<0.05 vs. $EPCR^{fl/fl}$ control littermate mice. Non-significant p values are not shown. Angiogenic sprout formation from aortic rings from $EPCR^{fl/fl}$ Tie2.Cre mice and $EPCR^{fl/fl}$ control littermates with and without DETA-NO (100 µM). (**B**) Representative findings and (**C**) the quantitative analysis are shown. ****p<0.0001 vs. $EPCR^{fl/fl}$ mice; ##p<0.01 vs. untreated $EPCR^{fl/fl}$ Tie2.Cre mice; n=3 biological replicates; One-Way ANOVA, Sidak's multiple comparisons test. Scale bars represent 100 µm.

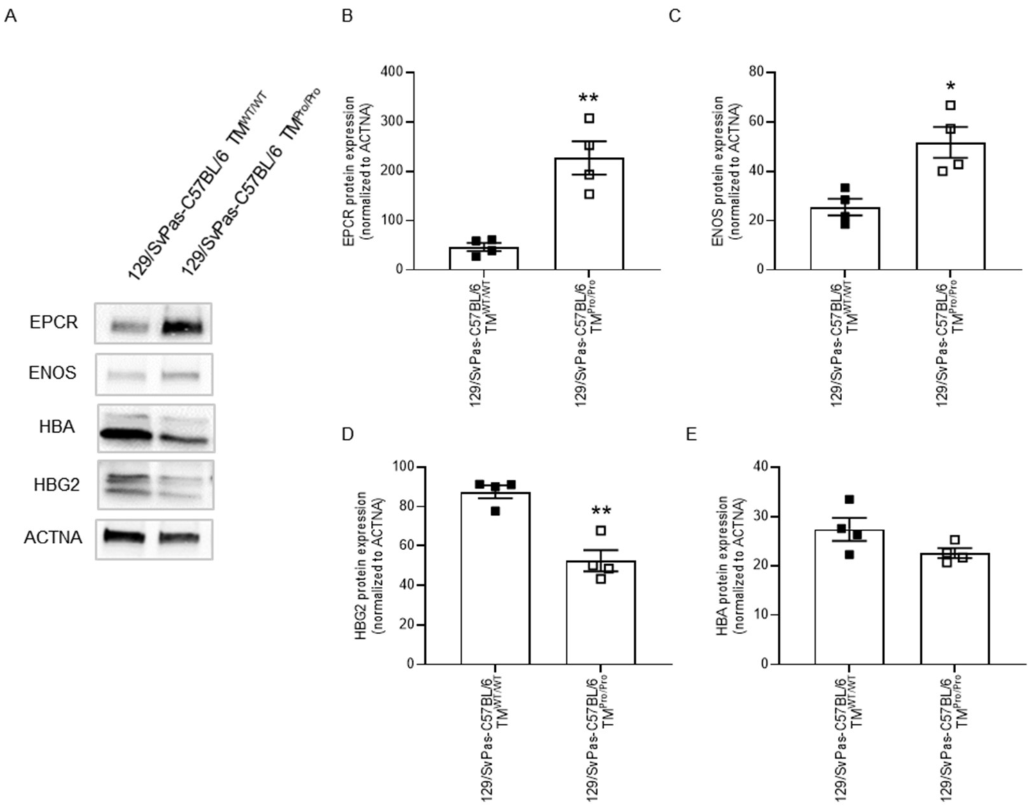

Supplemental Figure 12. EPCR-dependent eNOS and hemoglobin expression in endothelial cells. (**A**) Representative western blots and (**B**-**E**) quantitative analysis of primary endothelial cells isolated from 129/SvPas-C57BL/6 $TM^{Pro/Pro}$ mutant mice and their littermate TM^{WT} controls evaluated for (**B**) EPCR, (**C**) eNOS, (**D**) methemoglobin (HBG2) and (**E**) hemoglobin A (HBA) expression; n=4 biological replicates. *p<005 and **p<0.01 vs. control littermate mice using Student's t-test.

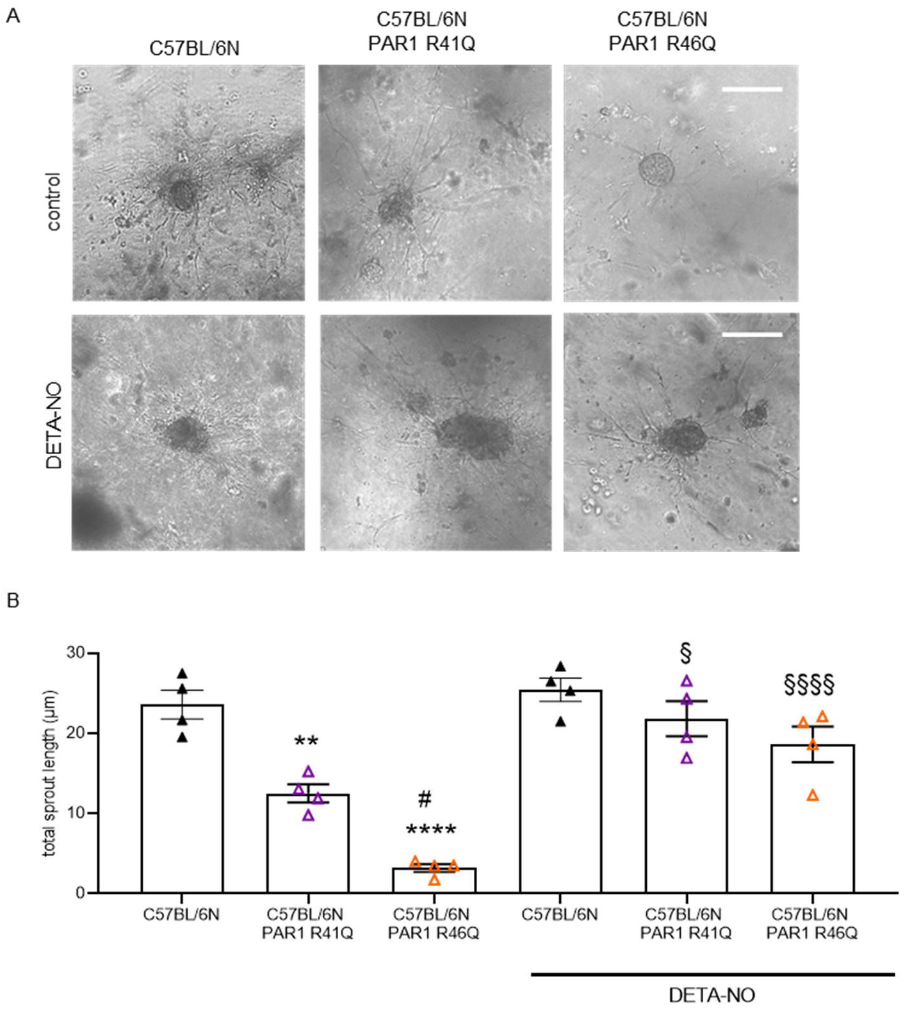

Supplemental Figure 13. Angiogenic sprouting of primary mouse endothelial cells isolated from mice with R41Q or R46Q point mutations in the PAR1 gene. (**A**) Representative microscopy brightfield images and (**B**) quantification of total sprout length in control (top panel) and DETA-NO treated (lower panel) primary endothelial cells isolated from C57BL/6N, C57BL/6N PAR1 R41Q and PAR1 R46Q mutant mice; n=4 biological replicates. **$p<0.01$ and ****$p<0.0001$ vs. C57BL/6N control mice; #$p<0.01$ vs. C57BL/6N PAR1 R41Q mice; §$p<0.05$ and §§§§$p<0.0001$ vs. untreated cells belonging to the same group; One-Way ANOVA, Sidak's multiple comparisons test.

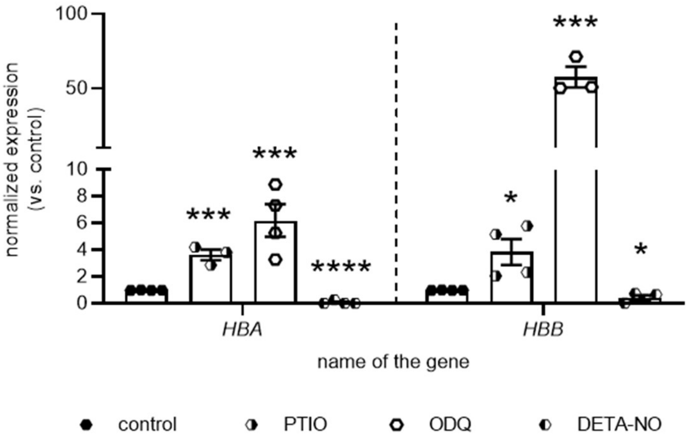

Supplemental Figure 14. Effects of modulation of NO availability and signaling on human endothelial cell hemoglobin expression. Human microvascular endothelial cells were treated with the NO scavenger PTIO (100 µM), the sGC inhibitor ODQ (50 µM) or the NO donor DETA-NO (100 µM) or equal volumes of DMSO (control) for four days and mRNA expression levels of hemoglobin isoform A (*HBA*) or isoform B (*HBB*) was determined using quantitative real-time PCR; n=3-4 biological replicates. Data were normalized to the housekeeping gene HPRT1 and are expressed as -fold change vs. control (set at 1). Results were analyzed using multiple t tests. *p<0.05, ***p<0.001 and ****p<0.0001 vs. control.

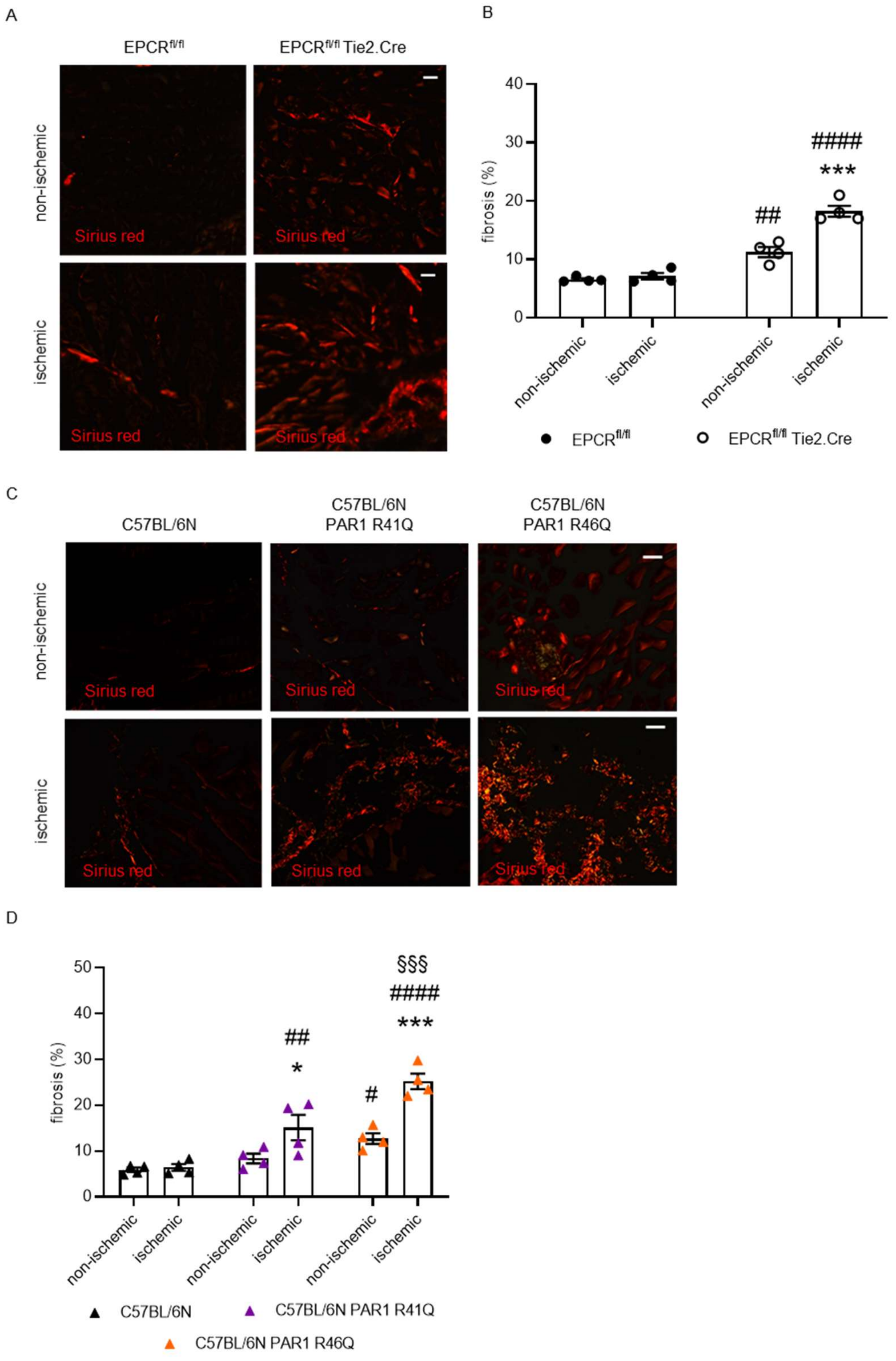

Supplemental Figure 15. Fibrotic postischemic remodeling in the presence of altered EPCR–aPC–PAR1 signaling. (**A**) Representative images (under polarized light) and (**B**) quantitative analysis after staining of ischemic and non-ischemic lower hindlimb muscles of EPCR$^{fl/fl}$ Tie2.Cre mice and EPCR$^{fl/fl}$ littermate controls or (**C** and **D**) C57BL/6N, C57BL/6N PAR1 R41Q and PAR1 R46Q mutant mice with Sirius Red (red); n=4 biological replicates; Two-Way ANOVA, Sidak's multiple comparisons test. *p<0.05 and ***p<0.001 vs. non-ischemic, contralateral hindlimb; ##p<0.01 and ####p<0.0001 vs. EPCR$^{fl/fl}$ or vs. C57BL/6N control mice, §§§p<0.001 vs. C57BL/6N PAR1 R41Q mice.

Supplemental Tables

Supplemental Table 1. Total erythrocyte numbers, hematocrit and hemoglobin levels in murine whole blood.

**Parameter**	**EPCR$^{fl/fl}$** (n=8)	**EPCR$^{fl/fl}$ Tie2.Cre** (n=8)	**C57BL/6N** (n=7)	**C57BL/6N PAR1$^{-/-}$** (n=6)
**RBC** (x10^6 per µL)	8.08 ± 0.3	7.75 ± 0.3	7.17 ± 0.3	7.48 ± 0.3
**HCT** (%)	43.1 ± 2.1	40.4 ± 1.4	37.1 ± 1.7	38.8 ± 0.7
**HGB** (g per dL)	12.8 ± 0.5	12.0 ± 0.5	11.7 ± 0.4	11.2 ± 0.3

Abbreviations: *HCT,* hematocrit; *HGB*, hemoglobin; *RBC*, red blood cells. Non-significant differences are not shown.

Supplemental Table 2. Baseline laboratory characteristics and markers of inflammation in patients with peripheral artery disease.

**Baseline laboratory characteristics**	
**Glucose** (mg/dL)	102 ± 3.3
**Triglycerides** (mg/dL)	161 ± 17
**Total cholesterol** (mg/dL)	193 ± 8.2
**LDL cholesterol** (mg/dL)	101 ± 7.3
**HDL cholesterol** (mg/dL)	59.1 ± 4.0
**CRP** (mg/dL)	2.5 ± 0.4
**WBC** (x 10^3/µL)	7.9 ± 0.3

The mean ± SEM are shown. Abbreviations: *CRP,* C-reactive protein; *HDL,* high density lipoprotein; *LDL,* low density lipoprotein; *WBC,* white blood cell count.

Supplemental Table 3. Functional capacities and medication in in patients with peripheral artery disease.

**Patients (n)**	19
**Lesion localization, left leg** (%)	8 (42.1)
**Lesion localization, right leg** (%)	0 (0)
**Lesion localization, both legs** (%)	11 (57.9)
**Functional capacities**	
**Absolute claudication distance** (m)	98 (85; 270)
**Ankle-brachial index**	0.78 ± 0.04
**Risk factors**	
**Arterial hypertension** (%)	16 (84.2)
**Diabetes mellitus** (%)	6 (31.6)
**Dyslipidemia** (%)	14 (73.7)
**Metabolic syndrome** (%)	8 (42.1)
**Family history** (%)	3 (15.8)
**Medication**	
**ACE inhibitors / AT1 antagonists** (%)	12 (63.2)
**Acetylsalicylic acid** (%)	17 (89.5)
**Beta-blocker** (%)	10 (52.6)
**Calcium antagonists** (%)	11 (57.9)
**Nitrates** (%)	0 (0)
**P2Y12 inhibitors** (%)	2 (10.5)
**Statins** (%)	15 (78.9)

The mean ± SEM or the median (and the interquartile range) are shown.

Supplemental Table 4. Mouse primer sequence and PCR conditions for genotyping.

**Gene**	**Primer sequences**<br>**(in 5‘ – 3‘ direction)**	**T_m**<br>**(°C)**	**cycles**	**ref**
Par1 **deletion**	F: AGCCTTGGCTCTGACTCTGAATGG<br>R: TGCACAGACGGACTAAGGACTTGG	59	35	$F2r^{tm1a(EUCOMM)Hmgu}$
Par1 **mutants**	F: GTCTGTGCAGGATCTGAACC<br>R: GCTTCTTGACCTTCATCCTC	59	35	(1)

Abbreviation: *Par1,* protease-activated receptor-1.

Supplemental Table 5. Mouse primer sequences and PCR conditions for QPCR analysis.

Gene	Primer sequences<br>(in 5‘ – 3‘ direction)	T_m<br>(°C)	cycles	ref
Par1	F: CCTATGAGCCAGCCAGAATC<br>R: TAGACTGCCCTACCCTCCAG	60	40	(2)
Procr	F: GTAGCCAAGACGCCTCAGAT<br>R: GATAGGGGTCGCGGAAGT	60	40	(3)

Abbreviation: *Par1,* protease-activated receptor-1, *Procr,* endothelial protein C receptor.

Supplemental Table 6. Human primer sequences and qRT-PCR conditions

Gene	Primer sequences<br>(in 5‘ – 3‘ direction)	T_m<br>(°C)	cycles	ref
ACTA2	F: GACAGCTACGTGGGTGACGAA<br>R: TTTTCCATGTCGTCCCAGTTG	60	40	(4)
HBA	F: ATGGTGCTGTCTCCTGCCGACAAG<br>R: TTAACGGTATTTGGAGGTCAGCACGG	60	40	(5)
HBB	F: ACTCCTGAGGAGAAGTCTGCCGTTAC<br>R: TTGTCACAGTGCAGCTCACTCAGTGTG	60	40	(6)
HPRT1	F: GTAATTGGTGGAGATGATCTCTCAACT<br>R: TGTTTTGCCAGTGTCAATTATATCTTC	60	40	(7)
PECAM1	F: AACAGTGTTGACATGAAGAGCC<br>R: TGTAAAACAGCACGTCATCCTT	60	40	(8)
PDGFRB	F: CATGGGGGTATGGTTTTGT<br>R: GTAAGGTGCCAACCTGCAA	60	40	(9)

Abbreviations: *ACTA2*, smooth muscle α-actin; *HBA*, hemoglobin subunit alpha; *HBB*, hemoglobin subunit beta; *HPRT1*, hypoxanthine-guanine phosphoribosyltransferase (housekeeping gene); *PECAM1*, platelet and endothelial cell adhesion molecule 1; *PDGFRB*, platelet derived growth factor receptor beta.

References

1. Sinha RK, Wang Y, Zhao Z, Xu X, Burnier L, Gupta N, et al. PAR1 biased signaling is required for activated protein C in vivo benefits in sepsis and stroke. *Blood.* 2018;131(11):1163-71.
2. Shin SJ, Hang HT, Thang BQ, Shimoda T, Sakamoto H, Osaka M, et al. Role of PAR1-Egr1 in the Initiation of Thoracic Aortic Aneurysm in Fbln4-Deficient Mice. *Arterioscler Thromb Vasc Biol.* 2020;40(8):1905-17.
3. Scaldaferri F, Sans M, Vetrano S, Graziani C, De Cristofaro R, Gerlitz B, et al. Crucial role of the protein C pathway in governing microvascular inflammation in inflammatory bowel disease. *J Clin Invest.* 2007;117(7):1951-60.
4. Wicks J, Haitchi HM, Holgate ST, Davies DE, and Powell RM. Enhanced upregulation of smooth muscle related transcripts by TGF beta2 in asthmatic (myo) fibroblasts. *Thorax.* 2006;61(4):313-9.
5. Saha D, Koli S, Patgaonkar M, and Reddy KV. Expression of hemoglobin-alpha and beta subunits in human vaginal epithelial cells and their functional significance. *PLoS One.* 2017;12(2):e0171084.
6. Newton DA, Rao KM, Dluhy RA, and Baatz JE. Hemoglobin is expressed by alveolar epithelial cells. *J Biol Chem.* 2006;281(9):5668-76.
7. Galiveti CR, Rozhdestvensky TS, Brosius J, Lehrach H, and Konthur Z. Application of housekeeping npcRNAs for quantitative expression analysis of human transcriptome by real-time PCR. *RNA.* 2010;16(2):450-61.
8. Behr B, Tang C, Germann G, Longaker MT, and Quarto N. Locally applied vascular endothelial growth factor A increases the osteogenic healing capacity of human adipose-derived stem cells by promoting osteogenic and endothelial differentiation. *Stem Cells.* 2011;29(2):286-96.
9. Yang J, Liu X, Nyland SB, Zhang R, Ryland LK, Broeg K, et al. Platelet-derived growth factor mediates survival of leukemic large granular lymphocytes via an autocrine regulatory pathway. *Blood.* 2010;115(1):51-60.
